# Developing a Gene Expression Model for Predicting Ventilator-Associated Pneumonia in Trauma Patients: A Pilot Study

**DOI:** 10.1371/journal.pone.0042065

**Published:** 2012-08-15

**Authors:** Joseph M. Swanson, G. Christopher Wood, Lijing Xu, Lisa E. Tang, Bernd Meibohm, Ramin Homayouni, Martin A. Croce, Timothy C. Fabian

**Affiliations:** 1 Department of Clinical Pharmacy, The University of Tennessee Health Science Center, Memphis, Tennessee, United States of America; 2 Department of Pharmaceutical Sciences, The University of Tennessee Health Science Center, Memphis, Tennessee, United States of America; 3 Department of Surgery, The University of Tennessee Health Science Center, Memphis, Tennessee, United States of America; 4 Department of Biological Sciences, The University of Memphis, Memphis, Tennessee, United States of America; 5 Bioinformatics Program, The University of Memphis, Memphis, Tennessee, United States of America; National Taiwan University Hospital, Taiwan

## Abstract

**Background:**

Ventilator-associated pneumonia (VAP) carries significant mortality and morbidity. Predicting which patients will become infected could lead to measures to reduce the incidence of VAP.

**Methodology/Principal Findings:**

The goal was to begin constructing a model for VAP prediction in critically-injured trauma patients, and to identify differentially expressed genes in patients who go on to develop VAP compared to similar patients who do not. Gene expression profiles of lipopolysaccharide stimulated blood cells in critically injured trauma patients that went on to develop ventilator-associated pneumonia (n = 10) was compared to those that never developed the infection (n = 10). Eight hundred and ten genes were differentially expressed between the two groups (ANOVA, P<0.05) and further analyzed by hierarchical clustering and principal component analysis. Functional analysis using Gene Ontology and KEGG classifications revealed enrichment in multiple categories including regulation of protein translation, regulation of protease activity, and response to bacterial infection. A logistic regression model was developed that accurately predicted critically-injured trauma patients that went on to develop VAP (VAP+) and those that did not (VAP−). Five genes (PIK3R3, ATP2A1, PI3, ADAM8, and HCN4) were common to all top 20 significant genes that were identified from all independent training sets in the cross validation. Hierarchical clustering using these five genes accurately categorized 95% of patients and PCA visualization demonstrated two discernable groups (VAP+ and VAP−).

**Conclusions/Significance:**

A logistic regression model using cross-validation accurately predicted patients that developed ventilator-associated pneumonia and should now be tested on a larger cohort of trauma patients.

## Introduction

Ventilator-associated pneumonia (VAP) is the most common serious infection in critically ill patients and results in significant morbidity, mortality, and health care costs [1]. Overall, 9–27% of mechanically ventilated patients develop VAP; however, trauma patients are at the highest risk [2]. Trauma-related risk factors for VAP have been identified, but a fundamental unanswered question is why some patients develop VAP while similar patients do not. A clinically useful tool to identify patients who are at risk for VAP would allow targeted prophylaxis. One method to determine which patients receive prophylaxis would be to establish the genetic profile that identifies patients more likely to develop VAP.

Previously, a number of single gene polymorphism studies have shown that over or under expression of immuno/inflammatory genes such as TNF-α, interleukin (IL)-1, IL-10, interferon gamma, and CD14 receptor are related to infection development [3].

However, none of these polymorphisms alone are sensitive or specific enough to be used to predict infections. It is highly unlikely that one polymorphism will be responsible for, or a suitable indicator of, infection risk.

Genome-wide screening approaches may be useful for identification of new genetic factors or gene expression profiles that are associated with infection development. cDNA microarrays can identify a broad range of differentially expressed genes in patients who develop infection compared to those who do not. These gene expression profiles may be used to predict infection risk. A focus on VAP is important because it is the most common serious infection in the intensive care unit (ICU). The purpose of this pilot study was to begin constructing a model for ventilator-associated pneumonia (VAP) prediction in critically-injured trauma patients, and to identify differentially expressed genes in patients who go on to develop VAP compared to similar patients who do not.

## Methods

### Patient enrollment

This study was performed at the Level 1 Presley Regional Trauma Center housed in the Regional Medical Center in Memphis, TN. Inclusion criteria were age 1 8–65 years, expected need for mechanical ventilation >6 days, and one of the following trauma-related risk factors for VAP [4]: severe traumatic brain injury (Glasgow coma score 4–8), severe thoracic trauma (multiple rib fractures or pulmonary contusions), spinal cord injury with paralysis, or a combination of injuries that placed the patient at risk for VAP as determined by the attending physician (e.g. severe intraabdominal trauma). Exclusion criteria were expected nonsurvivability of injuries, history of significant lung disease (e.g. COPD, asthma) immunocompromised state (e.g. pharmacologic, HIV infection), or pregnancy.

Patients were enrolled within 72 hours of trauma intensive care unit (TICU) admission. Patients were then followed throughout their ICU stay to monitor for the development of VAP, and were subsequently categorized at VAP+ or VAP−. VAP was definitively diagnosed using the center's standard criteria. Patients with fever/hypothermia (>38°C or <36°C), leukocytosis/leukopenia (>12,000/mm^3^ or <4,000/mm^3^), purulent sputum, and new or progressive infiltrate on chest radiograph underwent diagnostic bronchoscopic bronchoalveolar lavage (BAL) using a method previously described [5], [6]. A definitive diagnosis of VAP required growth of a pathogenic organism from the quantitative BAL culture ≥10^5^ colony forming units/mL. This diagnostic method is recommended by the ATS/IDSA guidelines (1). At design of this study, the optimal determination of study size for microarray studies had not been established. Therefore, the method by Simon et al. was used. A power analysis using the method by Simon et al. suggested a need for approximately 24 patients for this study [7]. Based on previous data, it was expected that the incidence of VAP would approach 50% in this population [8]. Thus, patients were expected to accrue into the VAP+ and VAP− groups in approximately equal fashion.

### Sample preparation

Upon enrollment, 40 mL of whole blood was collected. Blood samples were immediately stimulated with 1000 ng/mL of lipopolysaccharide (LPS) solution (E. coli 011B4 LPS in RPMI 1640 culture medium −10% fetal bovine serum and 100 U/mL penicillin-streptomycin) to approximate the effect of colonization with Gram-negative bacilli [9]. This model was used because the development of Gram-negative VAP is preceded by bacterial colonization. It was thought that the interpatient genetic variability in the immuno/inflammatory response to bacterial colonization would be important in determining which patients went on to develop VAP. Samples were incubated for 3 hours in a water bath at 37°C and then centrifuged for 10 minutes at 4°C. The plasma layer was decanted. The white blood cell layer was removed and incubated in 30 mL of RBC lysis buffer (Tris HCL/TRIZMA HCl/NH4Cl) at 37°C for 15 minutes then centrifuged for 15 minutes at 4°C. The supernatant was decanted and the pellet resuspended and washed with D-PBS two times.

Total RNA was isolated from peripheral blood using phenol-chloroform extraction per the RNAgents Total RNA isolation kit protocol (Promega, Madison, WI). RNA concentration was initially estimated by comparing the ultraviolet absorbance (A260/A280) ratio of the sample. The integrity and concentration of the RNA sample was subsequently assessed using the RNA 6000 Nano LabChip Kit and Agilent 2100 bioanalyzer (Agilent Technologies). Approximately 5 mcg of total RNA was used for cDNA synthesis. The MessageAmp aRNA kit (Ambion) was used for cDNA and cRNA synthesis. The labeled cRNA samples were then hybridized to the GeneChip Human Genome Focus Arrays (Affymetrix).

### Ethics

This study was approved by the University of Tennessee Health Science Center Institutional Review Board. The study was conducted in accordance with the Declaration of Helsinki. Informed consent was required from the patient or legally authorized representative. Once written consent was obtained the patient was enrolled into the study.

### Expression Analysis

All analysis was performed using Partek software (St Louis, MO). Differentially expressed genes were identified by ANOVA. Estimation of false discovery rate (FDR) due to multiple hypothesis testing was obtained by calculating the q-value as described by Storey and Tibshirani [10]. These genes were used to cluster patients by Hierarchical clustering using Pearson's dissimilarity scores and average linkage parameters. In addition, principal components analysis (PCA), which is a mathematical technique used to reduce the dimensionality of the data, was used to project patients in 3 dimensions based on their gene expression profiles. Lastly, patients were classified based on gene expression profiles using logistic regression model. VAP status prediction was obtained by fitting data to a logit function/logistic curve and calculating the posterior class probability. Leave-one-out partitioning cross-validation was used to evaluate the model accuracy. One round of cross-validation divided data into a training set (19 patients) and a test set (1 patient). The top 20 significant genes were selected from the training dataset based on all available genes on the chip using ANOVA. There were 20 rounds of cross-validation performed. The 20 correct rates were normalized to get the final accuracy.

Functional analysis of genes was performed using GO and KEGG annotations available through WebGestalt tool [11]. The p-values for enrichment of each category C, given our gene list A (containing n genes) and the reference gene list B (containing m genes) were calculated as follows. If there were k genes from A and j genes from B in a given category C, the expected number of genes in Category C can be calculated by 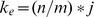. Therefore, the enrichment ratio is calculated by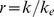. If k is greater than the expected number k_e_ (i.e. r is greater than 1), then category C is considered to be enriched in our gene list. The significance of the enrichment was calculated using the hypergeometric distribution, 
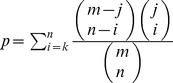
.

## Results

Thirty-two patients were enrolled within 72 hours of admission to the TICU, 12 patients could not be evaluated. Two patients died within 48 hours of admission, two patients were extubated within 48 hours of admission and samples in eight patients had insufficient quantities of RNA for the microarrays. Thus 20 patients were included, ten that went on to develop VAP (VAP+) and ten that did not (VAP−). Patients in the two groups were similar except with regard to duration of mechanical ventilation and length of ICU stay, which were significantly longer in the patients that developed VAP (Table 1).

**Table 1:** Patient Demographics.

Parameter	*VAP+ (n = 10)	*VAP− (n = 10)	p-value
Age (years)	35±16	35±15	NS
Sex (male/female)	8/3	7/3	NS
APACHE II	21±6	18±3	NS
Type of Injury (%):			
Severe TBI	64%	40%	NS
Thoracic trauma	64%	70%	NS
Spinal cord injury	0%	0%	
Abdominal trauma	55%	20%	NS
Mechanical ventilation (days)	17±9	8±4	0.007
ICU length of stay (days)	20±10	14±8	0.09
Hospital length of stay (days)	41±32	26±17	NS
Hospital mortality (%)	18%	0%	NS

* VAP+: Patients with ventilator-associated pneumonia; VAP−: Patients without ventilator associated pneumonia.

Data are presented as mean ± standard deviation, number, or percentages.

TBI: traumatic brain injury; ICU: intensive care unit.

A microarray approach was utilized to examine gene expression profiles in LPS treated blood cells from the VAP+ and VAP− groups. Using a one-way ANOVA test, 810 genes were identified whose transcript levels were significantly different between the two groups (Table S1). The q-value FDR estimates ranged from 0.035 to 0.44 (Table S1). This suggests that up to 44% of 810 genes (p<0.05) could be false positives. Although this number is quite high, it does not necessarily rule out the functional significance of the results. Indeed, functional analysis using Gene Ontology and KEGG classifications revealed several processes that were enriched among the differentially expressed genes (DEGs) (Table S2). For instance, a large number of DEGs are involved in regulation of protein translation (43 gene, p<6.98e^−08^), protein folding (21 genes, p<5e^−04^) and ribosomal machinery (28 genes, p<7.14e^−08^). Also, a set of DEGs are involved in regulation of protease activity: Serine peptidase (20 genes, p<2.2e^−03^), inhibitors of endopeptidases (18 genes, 2.3e^−03^), and inhibitor of metalloprotease activity (4 genes, p<4.00e^−03^). More importantly, a set of DEGs appeared to be involved in bacterial infection: antimicrobial humoral response (2 genes, p<8.80e^−03^), bacterial binding (5 genes, p<1.00e^−03^) and vibrio cholera infection (10 genes, 3.50e^−03^). These genes may contribute in some way to development of VAP.

**Figure 1:**
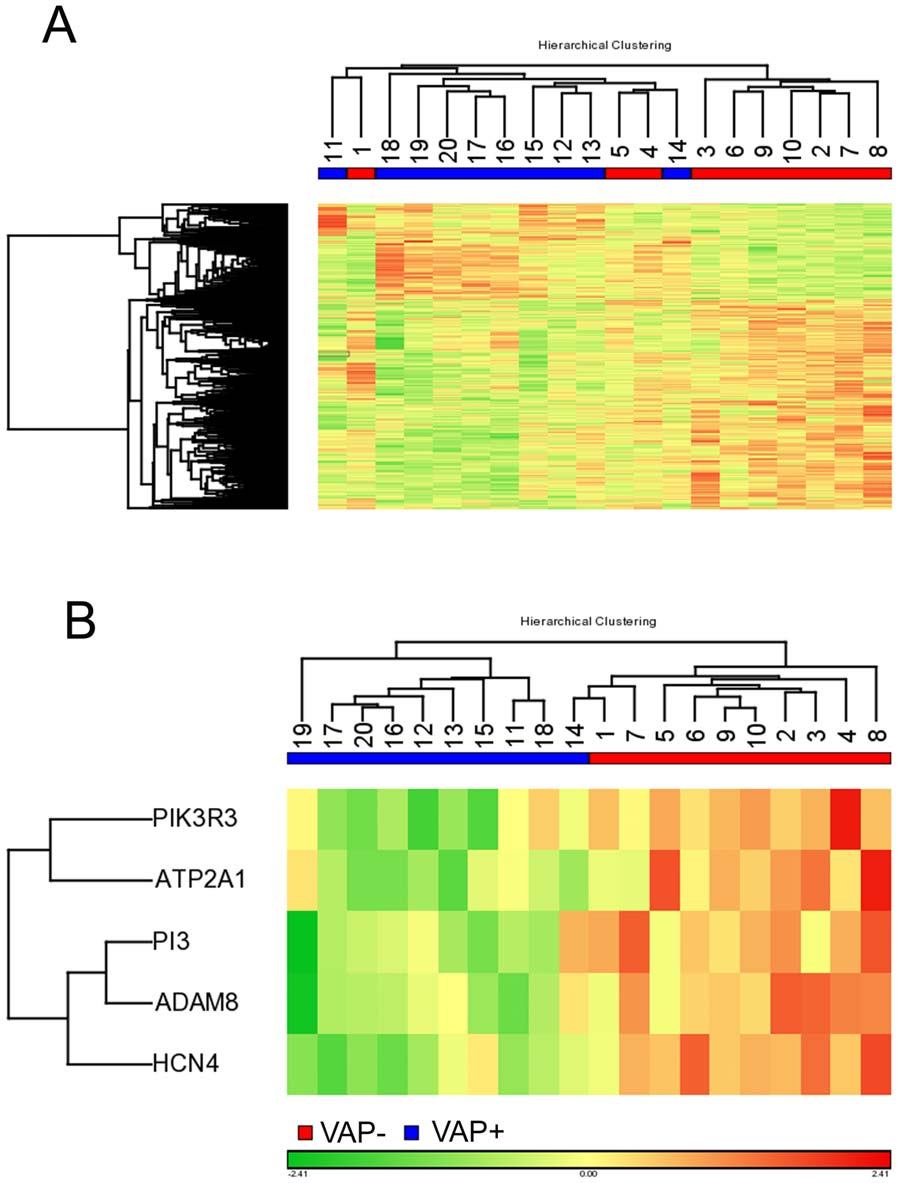
Hierarchical clustering of VAP− and VAP+ patients. (a) Hierarchical clustering of 810 differentially expressed genes in patients that went on to develop ventilator-associated pneumonia (blue) and those that did not (red). (b) Hierarchical clustering with the five genes that were common to all sets used in the cross validation tests.

**Figure 2:**
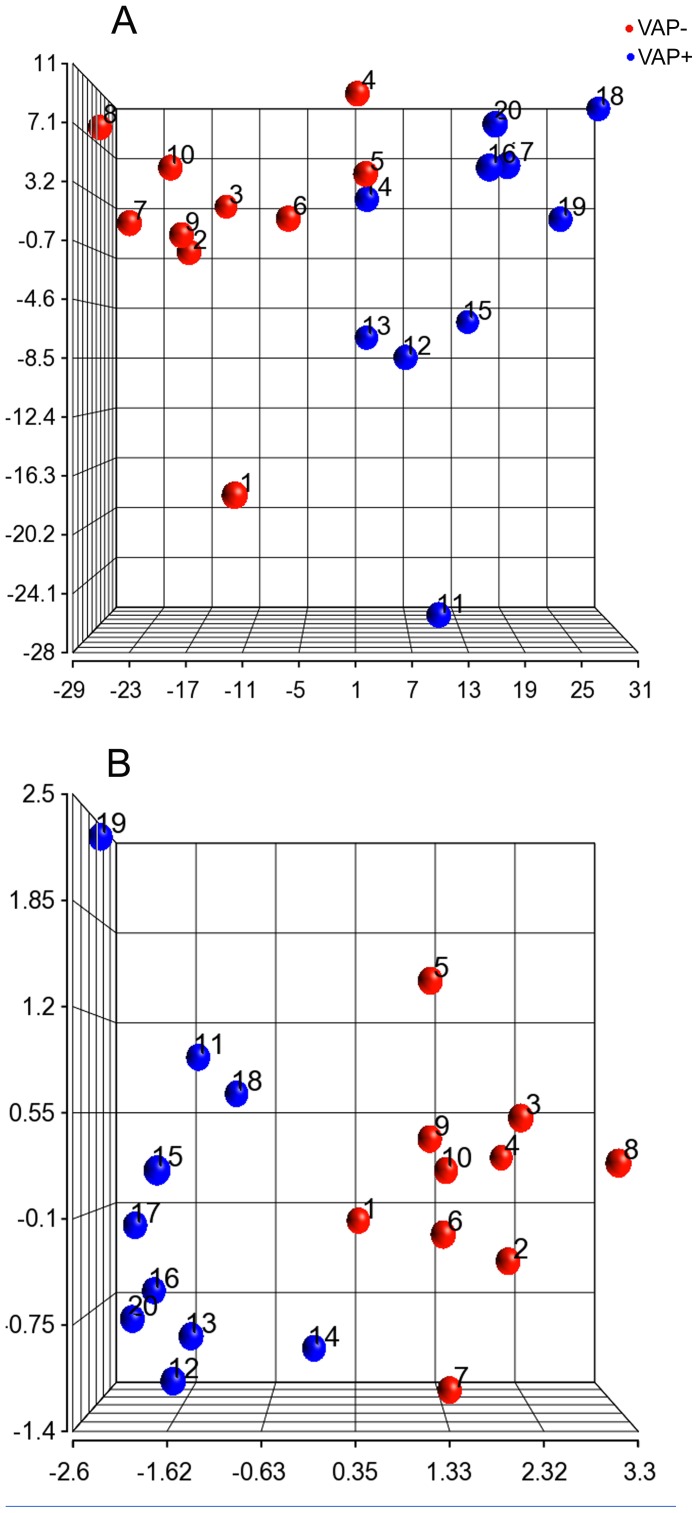
Principal component analysis for VAP− and VAP+ patients. (a) Sample clustering of patients that went on to develop ventilator-associated pneumonia (blue) and those that did not (red) using principal component analysis of 810 differentially expressed genes. (b) Sample clustering of patients that went on to develop ventilator-associated pneumonia (blue) and those that did not (red) using principal component analysis of 5 genes that were common to all sets in the cross validation tests.

Multiple approaches were used to determine if gene expression profiles could be used to cluster patients that go on to develop VAP. First, hierarchical clustering was performed using the gene expression levels of 810 DEGs identified by ANOVA (Figure 1A). Seventeen out of 20 patients' samples clustered according to their VAP classification, except for #1, #4 and #5 (VAP+). These results were consistent with the distribution of samples observed by principal component analysis (PCA), a mathematical method to reduce dimensionality of the data. Projection of the samples in the 3 largest components (accounting for approximately 59.5% of the variation in the 810 DEGs across the 20 samples) revealed that all patients except #1, #4 and #5 were segregating clearly in the two groups (Figure 2A).

To more rigorously test the predictive power of the gene expression profiles, a logistic regression model was implemented using a leave-one-out cross validation. Here, each sample was removed from the pool of 20 and DEGs were identified using a one-way ANOVA. The positive predictive value of the top 20 significant genes to classify patients as VAP+ or VAP− was then determined and reported as a posterior probability (Table 2). This model accurately predicted the VAP status of all patients except #15. The posterior probability of predicting VAP+ for VAP− patients ranged from 2.85e^−05^ to 0, with no false positives. Conversely, the posterior probability of predicting VAP− for VAP+ patients was 0 for all cases, except for one patient (#15), which could not be determined.

**Table 2:** Positive predictive value for ventilator-associated pneumonia using the expression profiles of the top five genes.

Patient Number	Correct Prediction	VAP− Posterior Probabilities	VAP+ Posterior Probabilities
* *VAP−:*			
1	Yes	1	2.88e^−22^
2	Yes	1	4.46e^−16^
3	Yes	0.999961495	3.85e^−05^
4	Yes	1	4.57e^−142^
5	Yes	1	0
6	Yes	1	1.21e^−13^
7	Yes	1	1.96e^−64^
8	Yes	1	0
9	Yes	0.999987634	1.24e^−05^
10	Yes	1	1.22e^−168^
* *VAP+:*			
11	Yes	0	1
12	Yes	0	1
13	Yes	0	1
14	Yes	0	1
15	No	0	1
16	Yes	0	1
17	Yes	0	1
18	Yes	0	1
19	Yes	0	1
20	Yes	0	1

* VAP+: Patients with ventilator-associated pneumonia; VAP−: Patients without ventilator associated pneumonia.

Five genes (PIK3R3, ATP2A1, PI3, ADAM8, and HCN4) were common to all significant gene sets used in the cross validation tests. After LPS stimulation of whole blood, the expression of these five genes were down regulated in patients' that went on to develop VAP (Figure 1B). To further validate the association of these genes with VAP status, PCA and hierarchical clustering analysis were repeated (Figure 1B and 2B). Again, expression profiles of these 5 genes accurately clustered all patients except sample 14 (Figure 1B). Two groups were discernible by PCA visualization except patient samples 1 and 14, which appeared to be at the interface (Figure 2B).

## Discussion

The key result of this pilot study was development of a logistic regression model that accurately predicted critically-injured trauma patients that went on to develop VAP (VAP+) and those that did not (VAP−). Five genes (PIK3R3, ATP2A1, PI3, ADAM8, and HCN4) were common to all top 20 significant genes which were identified from all independent training sets in the cross validation. This model is supported by the fact that hierarchical clustering using these five genes accurately categorized 95% of patients and PCA visualization demonstrated two discernable groups, VAP+ and VAP−. This preliminary research is the first step in developing a clinically useful screening tool to identify patients at highest genetic risk of VAP secondary to trauma and subsequent bacterial exposure.

Of the five genes identified, three appear to have a clear role in host response to infection. Since all five genes were down regulated in response to LPS stimulation, it is logical that they may play a role in the relative susceptibility to infection. PI3 has been best studied in the host response to infections. It encodes for an elastase-specific inhibitor with antimicrobial peptide activity that is synthesized in response to cytokine and bacterial stimuli [12]. It has been shown to promote early clearance of *Pseudomonas aeruginosa* via macrophage activation and neutrophil recruitment [13]. ADAM8 encodes a distintegrin and metalloproteinase domain-containing protein 8. Neutrophil activation by proinflammatory cytokines induces rapid translocation of ADAM8 to the cell membrane with subsequent shedding [14]. This process is associated with shedding of L-selectin, which is intimately tied to transendothelial migration of neutrophils [15]. PIK3R3 encodes for phosphoinositide 3-kinase regulatory subunit gamma (PI3Kγ), which is part of a large family of enzymes involved in intracellular signaling. PI3Kγ is predominately expressed immune cells and plays a role in chemoattractant-induced cell migration [16], [17], [18], [19]. Thus, down regulation of these genes following LPS stimulation would seem to place the host at a greater risk of developing an infection.

The role of the remaining two genes in response to infection is less clear. HCN4 codes for the hyperpolarization activated cyclic nucleotide-gated potassium channel. Its role in cardiac rhythm maintenance is well defined [20], but there are currently no data linking it to infectious complications of critically ill patients. ATP2A1 encodes the sarcoplasmic reticulum calcium transporting ATPase (SERCA1) in the of skeletal muscle [21]. It is interesting to note that LPS can cause myocardial dysfunction in critically-ill patients and that SERCA is intimately related to cardiac relaxation [22]. These two processes could be related and may be a reason why we found ATP2A1 was down regulated in VAP+ patients after LPS stimulation. While the role of these two genes in infections are not as clear, they were still important to the model as they were among the top 20 DEGs identified for every leave-one-out cross validation. This consistency is noteworthy and could represent a relation to VAP in critically-injured trauma patients that has yet to be identified.

A number of previous studies suggest that genetic variability in infection risk exists in hospitalized patients. The most widely studied polymorphism is associated with overproduction of TNF-α; which has been related to a 2.1–13 fold increase in the incidence of severe sepsis from all causes including pneumonia [23]. Similar data exist on a smaller scale for IL-1, IL-10, interferon gamma, and CD14 receptor genes [24], [25], [26], [27]. However, none of these associations were sensitive or specific enough to be used as clinical tests for an increased risk of infection. As such, the current study design is different because it used gene expression profiling to search for candidate genes that may be useful clinical markers of infection risk.

Changes in gene expression patterns have been used in the early diagnosis of VAP [28], [29]. McDunn et al. identified 85 genes in a riboleukogram that identified patients with VAP at 24 to 96 hours before the clinical diagnosis could be established. These data demonstrate that differences in gene expression occur well before patients manifest clinical signs of infection. Cobb et al. validated the early VAP diagnosis using the riboleukogram, and the studies together support the concept that gene expression is a useful clinical diagnostic tool. It is reasonable that the genes identified in both studies relate to immune response, neutrophil activation, and intracellular signaling pathways. The current study is similar in that the genes identified in the logistic regression model also relate to the immune response and involve cell signaling (PIK3R3), antimicrobial peptides (PI3), and cell adhesion (ADAM8). The current study is different in that the logistic regression model developed does not diagnose pneumonia, but instead identifies patients at future risk of VAP. The potential benefit of these results would be the ability to identify at-risk patients upon ICU admission. The ultimate goal would be to use that information to employ prophylactic measures such as systemic and/or topical (i.e. gut, aerosolized) antibiotics to prevent VAP. Prophylaxis is highly effective and would benefit patients by reducing VAP-associated morbidity and mortality. Only targeting those at risk would reduce the likelihood for development of bacterial resistance, as this has been the main reason for avoiding prophylactic antibiotic therapies.

In addition to providing a predictive tool, gene expression profiling can also provide new insights into the mechanism of the disease. We found 810 genes, which were differentially regulated in VAP patients, compared to controls. Unfortunately, the q-value FDR estimates were as high as 44% for some genes (Table S1). This makes it difficult to determine if a given gene might have a specific role in VAP development. In spite of this shortcoming, we found that using the 810 gene expression profiles, the samples could be delineated fairly accurately by hierarchical clustering or by PCA visualization, which gives us some confidence that the DEGs might have some functional relevance. Indeed, we noted several sets of functionally related genes that were affected similarly in VAP patients. For example all 25 genes in ‘translational elongation’ category and both genes in ‘antimicrobial humoral response’ category were down regulated in VAP+ patients. On the other hand, all five genes in ‘bacterial binding’ category were up regulated in VAP+ patients. In addition, a large number of genes involved in regulation of endopeptidase activities were differentially regulated in VAP+ patients. Taken together, these results suggest that these processes may play an important role in development of VAP.

### Limitations

The current study has limitations. First, the pilot nature of the study is hypothesis generating and requires further validation of the findings. Second, the predictive model was developed with 20 patients and was not tested on an independent sample of patients. While these issues limit the immediate clinical value of this study, the stage is set to test the predictive value of the five genes identified across a larger patient cohort. Third, the timeframe for enrollment was large, within the first 72 hours following trauma intensive care unit admission. It is likely that time from injury results in changes in inflammation and gene expression. However, the optimal time to relate gene expression to future infectious processes has not been established. Future studies may benefit from a shorter time window for enrollment. Fourth, as mentioned above, due to multiple hypothesis testing the FDR reported for the 810 genes was high. However, it is important to note that this result does not impact the identification of predictive genes by an independent regression model, which included a leave-one-out cross validation procedure. Finally, performing LPS stimulation prior to measuring gene expression is relatively uncommon. This method was used in the current study to provide a gene expression response that would approximate the in vivo response in patients exposed to Gram-negative bacteria. In designing the study, the investigators believed that this would better accentuate the true genetic differences between those who go on to develop VAP compared to those who do not. A recent study by Bryant et al. found that LPS stimulation produced variable expression in immune-related genes. The authors concluded that this variability may be related to interpatient differences in response to an infectious insult [30]. Additionally, Textoris J, et al. analyzed unstimulated whole blood and failed to see gene expression differences in trauma patients that developed VAP and those that did not [31].

This small pilot study has three important strengths. First, the definition of VAP used in the study was the most rigorous diagnosis available, and is considered to be an optimal diagnosis by the current ATS/IDSA guidelines [1]. Thus, there is confidence that the patients in the study group actually had VAP and the control patients did not. Second, the use of the Affymetrix platform provides external validity. This is the most widely used microarray platform and the chip used in this study is widely available. Third, this was a relatively homogenous population of critically injured trauma patients with similar demographics between the VAP+ and VAP− groups.

## Conclusion

In this pilot study a logistic regression model using cross-validation accurately predicted patients that went on to develop ventilator-associated pneumonia. This model should now be tested on a larger cohort of trauma patients to validate its prognostic value.

## Supporting Information

Table S1 Differentially Expressed Genes. Using a one-way ANOVA test, 810 genes were differentially expressed in the VAP− and VAP+ groups. The q-value FDR estimates ranged from 0.035 to 0.44.
(DOC)

Table S2 Significantly enriched Gene Ontology categories. This table contains only the most distal (specific) categories in a particular ontology. The number of genes within each category that are either up- or down-regulated in VAP+ patients is indicated. For a complete listing of all enriched GO categories, please see Table S1.
(DOC)

## References

[1] Guidelines for the management of adults with hospital-acquired, ventilator-associated, and healthcare-associated pneumonia. Am J Respir Crit Care Med 171: 388–416. 15699079 10.1164/rccm.200405-644ST

[2] Hedrick TL, Smith RL, McElearney ST, Evans HL, Smith PW, et al (2008) Differences in early- and late-onset ventilator-associated pneumonia between surgical and trauma patients in a combined surgical or trauma intensive care unit. J Trauma 64: 714–720. 18332812 10.1097/TA.0b013e31811ec18e

[3] Namath A, Patterson AJ (2009) Genetic polymorphisms in sepsis. Crit Care Clin 25: 835–856. 19892256 10.1016/j.ccc.2009.06.004

[4] Croce MA, Fabian TC, Waddle-Smith L, Maxwell RA (2001) Identification of early predictors for post-traumatic pneumonia. Am Surg 67: 105–110. 11243529

[5] Croce MA, Fabian TC, Waddle-Smith L, Melton SM, Minard G, et al (1998) Utility of Gram's stain and efficacy of quantitative cultures for posttraumatic pneumonia: a prospective study. Ann Surg 227: 743–751 discussion 751-745. 9605666 10.1097/00000658-199805000-00015 PMC1191359

[6] Swanson JM, Wood GC, Croce MA, Mueller EW, Boucher BA, et al (2008) Utility of preliminary bronchoalveolar lavage results in suspected ventilator-associated pneumonia. J Trauma 65: 1271–1277. 19077612 10.1097/TA.0b013e3181574d6a

[7] Simon R, Radmacher MD, Dobbin K (2002) Design of studies using DNA microarrays. Genet Epidemiol 23: 21–36. 12112246 10.1002/gepi.202

[8] Wood GC, Boucher BA, Croce MA, Hanes SD, Herring VL, et al (2002) Aerosolized ceftazidime for prevention of ventilator-associated pneumonia and drug effects on the proinflammatory response in critically ill trauma patients. Pharmacotherapy 22: 972–982. 12173800 10.1592/phco.22.12.972.33596

[9] Heagy W, Nieman K, Hansen C, Cohen M, Danielson D, et al (2003) Lower levels of whole blood LPS-stimulated cytokine release are associated with poorer clinical outcomes in surgical ICU patients. Surg Infect (Larchmt) 4: 171–180. 12906717 10.1089/109629603766956960

[10] Storey JD, Tibshirani R (2003) Statistical significance for genomewide studies. Proceedings of the National Academy of Sciences of the United States of America 100: 9440–9445. 12883005 10.1073/pnas.1530509100 PMC170937

[11] Zhang B, Kirov S, Snoddy J (2005) WebGestalt: an integrated system for exploring gene sets in various biological contexts. Nucleic Acids Res 33: W741–748. 15980575 10.1093/nar/gki475 PMC1160236

[12] Sallenave JM, Shulmann J, Crossley J, Jordana M, Gauldie J (1994) Regulation of secretory leukocyte proteinase inhibitor (SLPI) and elastase-specific inhibitor (ESI/elafin) in human airway epithelial cells by cytokines and neutrophilic enzymes. Am J Respir Cell Mol Biol 11: 733–741. 7946401 10.1165/ajrcmb.11.6.7946401

[13] Wilkinson TS, Dhaliwal K, Hamilton TW, Lipka AF, Farrell L, et al (2009) Trappin-2 promotes early clearance of Pseudomonas aeruginosa through CD14-dependent macrophage activation and neutrophil recruitment. Am J Pathol 174: 1338–1346. 19264904 10.2353/ajpath.2009.080746 PMC2671365

[14] Gomez-Gaviro M, Dominguez-Luis M, Canchado J, Calafat J, Janssen H, et al (2007) Expression and regulation of the metalloproteinase ADAM-8 during human neutrophil pathophysiological activation and its catalytic activity on L-selectin shedding. J Immunol 178: 8053–8063. 17548643 10.4049/jimmunol.178.12.8053

[15] Smalley DM, Ley K (2005) L-selectin: mechanisms and physiological significance of ectodomain cleavage. J Cell Mol Med 9: 255–266. 15963248 10.1111/j.1582-4934.2005.tb00354.x PMC6740228

[16] Deane JA, Fruman DA (2004) Phosphoinositide 3-kinase: diverse roles in immune cell activation. Annu Rev Immunol 22: 563–598. 15032589 10.1146/annurev.immunol.22.012703.104721

[17] Ruckle T, Schwarz MK, Rommel C (2006) PI3Kgamma inhibition: towards an ‘aspirin of the 21st century’? Nat Rev Drug Discov 5: 903–918. 17080027 10.1038/nrd2145

[18] Ferrandi C, Ardissone V, Ferro P, Ruckle T, Zaratin P, et al (2007) Phosphoinositide 3-kinase gamma inhibition plays a crucial role in early steps of inflammation by blocking neutrophil recruitment. J Pharmacol Exp Ther 322: 923–930. 17526805 10.1124/jpet.107.123026

[19] Sasaki T, Suzuki A, Sasaki J, Penninger JM (2002) Phosphoinositide 3-kinases in immunity: lessons from knockout mice. J Biochem 131: 495–501. 11926985 10.1093/oxfordjournals.jbchem.a003126

[20] Baruscotti M, Bottelli G, Milanesi R, DiFrancesco JC, DiFrancesco D (2010) HCN-related channelopathies. Pflugers Arch 460: 405–415. 20213494 10.1007/s00424-010-0810-8

[21] Hovnanian A (2007) SERCA pumps and human diseases. Subcell Biochem 45: 337–363. 18193643 10.1007/978-1-4020-6191-2_12

[22] Heitner SB, Hollenberg SM (2009) The cardiac force-frequency relationship and frequency-dependent acceleration of relaxation are impaired in lipopolysaccharide-treated rats: is the phospholamban-SERCA axis a therapeutic target? Crit Care 13: 132. 19439042 10.1186/cc7752 PMC2689476

[23] O'Dwyer MJ, Mankan AK, Stordeur P, O'Connell B, Duggan E, et al (2006) The occurrence of severe sepsis and septic shock are related to distinct patterns of cytokine gene expression. Shock 26: 544–550. 17117127 10.1097/01.shk.0000235091.38174.8d

[24] Ma P, Chen D, Pan J, Du B (2002) Genomic polymorphism within interleukin-1 family cytokines influences the outcome of septic patients. Crit Care Med 30: 1046–1050. 12006801 10.1097/00003246-200205000-00015

[25] Stassen NA, Leslie-Norfleet LA, Robertson AM, Eichenberger MR, Polk HC Jr (2002) Interferon-gamma gene polymorphisms and the development of sepsis in patients with trauma. Surgery 132: 289–292. 12219025 10.1067/msy.2002.127167

[26] Lowe PR, Galley HF, Abdel-Fattah A, Webster NR (2003) Influence of interleukin-10 polymorphisms on interleukin-10 expression and survival in critically ill patients. Crit Care Med 31: 34–38. 12544990 10.1097/00003246-200301000-00005

[27] Heesen M, Bloemeke B, Schade U, Obertacke U, Majetschak M (2002) The −260 C→T promoter polymorphism of the lipopolysaccharide receptor CD14 and severe sepsis in trauma patients. Intensive Care Med 28: 1161–1163. 12185442 10.1007/s00134-002-1389-0

[28] Cobb JP, Moore EE, Hayden DL, Minei JP, Cuschieri J, et al (2009) Validation of the riboleukogram to detect ventilator-associated pneumonia after severe injury. Ann Surg 250: 531–539. 19730236 10.1097/SLA.0b013e3181b8fbd5 PMC3047595

[29] McDunn JE, Husain KD, Polpitiya AD, Burykin A, Ruan J, et al (2008) Plasticity of the systemic inflammatory response to acute infection during critical illness: development of the riboleukogram. PLoS One 3: e1564. 18270561 10.1371/journal.pone.0001564 PMC2215774

[30] Bryant PA, Smyth GK, Robins-Browne R, Curtis N (2011) Technical variability is greater than biological variability in a microarray experiment but both are outweighed by changes induced by stimulation. PLoS One 6: e19556. 21655321 10.1371/journal.pone.0019556 PMC3104982

[31] Textoris J, Loriod B, Benayoun L, Gourraud PA, Puthier D, et al (2011) An evaluation of the role of gene expression in the prediction and diagnosis of ventilator-associated pneumonia. Anesthesiology 115: 344–352. 21796056 10.1097/ALN.0b013e318225ba26

