# Supplementary material for: Developing a Gene Expression Model for Predicting Ventilator-Associated Pneumonia in Trauma Patients: A Pilot Study
Source: PLoS One. 2012 Aug 15;7(8):e42065. doi: 10.1371/journal.pone.0042065 (PMC3419717; doi:10.1371/journal.pone.0042065)
Supplement: Table S1 — Differentially Expressed Genes. Using a one-way ANOVA test, 810 genes were differentially expressed in the VAP− and VAP+ groups. The q-value FDR estimates ranged from 0.035 to 0.44. (DOC) [file pone.0042065.s001.doc]

Table S1. Differentially Expressed Genes

| **Probeset ID** | **Gene Symbol** | **Gene Title** | **p-value**  **(VAP- vs. VAP+)** | **q-value** | **Fold-Change (VAP- vs. VAP+)** | **Fold-Change (Description)** |
| --- | --- | --- | --- | --- | --- | --- |
| 206946_at | HCN4 | hyperpolarization activated cyclic nucleotide-gated potassium channel 4 | 4.86E-06 | 0.0345552 | 1.23073 | VAP- up vs VAP+ |
| 205180_s_at | ADAM8 | ADAM metallopeptidase domain 8 | 0.000108873 | 0.290164 | 1.40526 | VAP- up vs VAP+ |
| 202743_at | PIK3R3 | phosphoinositide-3-kinase, regulatory subunit 3 (gamma) | 0.00015727 | 0.290164 | 1.14484 | VAP- up vs VAP+ |
| 205444_at | ATP2A1 | ATPase, Ca++ transporting, cardiac muscle, fast twitch 1 | 0.000238632 | 0.290164 | 1.1514 | VAP- up vs VAP+ |
| 41469_at | PI3 | peptidase inhibitor 3, skin-derived | 0.000262653 | 0.290164 | 1.9032 | VAP- up vs VAP+ |
| 218681_s_at | SDF2L1 | stromal cell-derived factor 2-like 1 | 0.000410887 | 0.290164 | 1.39808 | VAP- up vs VAP+ |
| 218405_at | ABT1 | activator of basal transcription 1 | 0.000458932 | 0.290164 | 1.27729 | VAP- up vs VAP+ |
| 201871_s_at | UBXN1 | UBX domain protein 1 | 0.000466714 | 0.290164 | 1.29024 | VAP- up vs VAP+ |
| 209270_at | LAMB3 | laminin, beta 3 | 0.000497191 | 0.290164 | 2.01511 | VAP- up vs VAP+ |
| 36920_at | MTM1 | myotubularin 1 | 0.000537356 | 0.290164 | -1.12263 | VAP- down vs VAP+ |
| 214270_s_at | MAPRE3 | microtubule-associated protein, RP/EB family, member 3 | 0.000542452 | 0.290164 | -1.28832 | VAP- down vs VAP+ |
| 205557_at | BPI | bactericidal/permeability-increasing protein | 0.000584123 | 0.290164 | -2.01435 | VAP- down vs VAP+ |
| 208034_s_at | PROZ | protein Z, vitamin K-dependent plasma glycoprotein | 0.000603716 | 0.290164 | 1.17397 | VAP- up vs VAP+ |
| 221904_at | FAM131A | family with sequence similarity 131, member A | 0.000621364 | 0.290164 | 1.4123 | VAP- up vs VAP+ |
| 204743_at | TAGLN3 | transgelin 3 | 0.000632843 | 0.290164 | 1.19695 | VAP- up vs VAP+ |
| 204385_at | KYNU | kynureninase (L-kynurenine hydrolase) | 0.000725278 | 0.290164 | 1.38124 | VAP- up vs VAP+ |
| 222047_s_at | SRRT | serrate RNA effector molecule homolog (Arabidopsis) | 0.000805568 | 0.290164 | 1.40505 | VAP- up vs VAP+ |
| 204542_at | ST6GALNAC2 | ST6 (alpha-N-acetyl-neuraminyl-2,3-beta-galactosyl-1,3)-N-acetylgalactosaminide | 0.000806382 | 0.290164 | -1.39088 | VAP- down vs VAP+ |
| 212955_s_at | POLR2I | polymerase (RNA) II (DNA directed) polypeptide I, 14.5kDa | 0.000850766 | 0.290164 | 1.24038 | VAP- up vs VAP+ |
| 202317_s_at | UBE4B | ubiquitination factor E4B (UFD2 homolog, yeast) | 0.000898708 | 0.290164 | -1.15871 | VAP- down vs VAP+ |
| 202418_at | YIF1A | Yip1 interacting factor homolog A (S. cerevisiae) | 0.000931112 | 0.290164 | 1.22894 | VAP- up vs VAP+ |
| 218502_s_at | TRPS1 | trichorhinophalangeal syndrome I | 0.00102993 | 0.290164 | -1.11875 | VAP- down vs VAP+ |
| 202135_s_at | ACTR1B | ARP1 actin-related protein 1 homolog B, centractin beta (yeast) | 0.00107994 | 0.290164 | 1.16641 | VAP- up vs VAP+ |
| 207352_s_at | GABRB2 | gamma-aminobutyric acid (GABA) A receptor, beta 2 | 0.00116184 | 0.290164 | -1.22686 | VAP- down vs VAP+ |
| 204398_s_at | EML2 | echinoderm microtubule associated protein like 2 | 0.00121689 | 0.290164 | 1.37047 | VAP- up vs VAP+ |
| 214373_at | --- | --- | 0.00125336 | 0.290164 | 1.19057 | VAP- up vs VAP+ |
| 205698_s_at | MAP2K6 | mitogen-activated protein kinase kinase 6 | 0.00125596 | 0.290164 | -1.4962 | VAP- down vs VAP+ |
| 206353_at | COX6A2 | cytochrome c oxidase subunit VIa polypeptide 2 | 0.00127865 | 0.290164 | 1.135 | VAP- up vs VAP+ |
| 221631_at | CACNA1I | calcium channel, voltage-dependent, T type, alpha 1I subunit | 0.00134045 | 0.290164 | 1.1785 | VAP- up vs VAP+ |
| 218336_at | PFDN2 | prefoldin subunit 2 | 0.00136663 | 0.290164 | 1.52862 | VAP- up vs VAP+ |
| 202388_at | RGS2 | regulator of G-protein signaling 2, 24kDa | 0.00140585 | 0.290164 | 1.67659 | VAP- up vs VAP+ |
| 221004_s_at | ITM2C | integral membrane protein 2C | 0.00140789 | 0.290164 | 1.23108 | VAP- up vs VAP+ |
| 203725_at | GADD45A | growth arrest and DNA-damage-inducible, alpha | 0.00141978 | 0.290164 | -1.65242 | VAP- down vs VAP+ |
| 204689_at | HHEX | hematopoietically expressed homeobox | 0.00148606 | 0.290164 | -1.43424 | VAP- down vs VAP+ |
| 202825_at | SLC25A4 | solute carrier family 25 (mitochondrial carrier; adenine nucleotide translocator | 0.0014975 | 0.290164 | 1.15657 | VAP- up vs VAP+ |
| 202858_at | U2AF1 | U2 small nuclear RNA auxiliary factor 1 | 0.00150125 | 0.290164 | 1.19748 | VAP- up vs VAP+ |
| 206468_s_at | METTL13 | methyltransferase like 13 | 0.00152842 | 0.290164 | 1.20993 | VAP- up vs VAP+ |
| 204334_at | KLF7 | Kruppel-like factor 7 (ubiquitous) | 0.00158503 | 0.290164 | -1.7501 | VAP- down vs VAP+ |
| 208837_at | TMED3 | transmembrane emp24 protein transport domain containing 3 | 0.00159087 | 0.290164 | 1.1922 | VAP- up vs VAP+ |
| 200019_s_at | FAU | Finkel-Biskis-Reilly murine sarcoma virus (FBR-MuSV) ubiquitously expressed | 0.00169453 | 0.296329 | 1.35975 | VAP- up vs VAP+ |
| 220048_at | EDAR | ectodysplasin A receptor | 0.00170799 | 0.296329 | 1.16203 | VAP- up vs VAP+ |
| 202328_s_at | PKD1 | polycystic kidney disease 1 (autosomal dominant) | 0.00189033 | 0.309457 | 1.2445 | VAP- up vs VAP+ |
| 205550_s_at | BRE | brain and reproductive organ-expressed (TNFRSF1A modulator) | 0.00190713 | 0.309457 | -1.22661 | VAP- down vs VAP+ |
| 219760_at | LIN7B | lin-7 homolog B (C. elegans) | 0.00191417 | 0.309457 | 1.21906 | VAP- up vs VAP+ |
| 221270_s_at | QTRT1 | queuine tRNA-ribosyltransferase 1 | 0.0019839 | 0.313089 | 1.25312 | VAP- up vs VAP+ |
| 201666_at | TIMP1 | TIMP metallopeptidase inhibitor 1 | 0.00202466 | 0.313089 | 1.3419 | VAP- up vs VAP+ |
| 204945_at | PTPRN | protein tyrosine phosphatase, receptor type, N | 0.00211227 | 0.319687 | 1.12482 | VAP- up vs VAP+ |
| 205513_at | TCN1 | transcobalamin I (vitamin B12 binding protein, R binder family) | 0.00219558 | 0.325373 | -1.61087 | VAP- down vs VAP+ |
| 207760_s_at | NCOR2 | nuclear receptor co-repressor 2 | 0.00234933 | 0.33523 | 1.73987 | VAP- up vs VAP+ |
| 209150_s_at | TM9SF1 | transmembrane 9 superfamily member 1 | 0.00248376 | 0.33523 | -1.18669 | VAP- down vs VAP+ |
| 206398_s_at | CD19 | CD19 molecule | 0.00250715 | 0.33523 | 1.22178 | VAP- up vs VAP+ |
| 219920_s_at | GMPPB | GDP-mannose pyrophosphorylase B | 0.00251899 | 0.33523 | 1.26181 | VAP- up vs VAP+ |
| 219317_at | POLI | polymerase (DNA directed) iota | 0.00260998 | 0.33523 | -1.15699 | VAP- down vs VAP+ |
| 201612_at | ALDH9A1 | aldehyde dehydrogenase 9 family, member A1 | 0.00262744 | 0.33523 | -1.3269 | VAP- down vs VAP+ |
| 212706_at | LOC100286937 /// LOC100287164 /// RASA4 | similar to HSPC047 protein /// similar to HSPC047 protein /// RAS p21 protein ac | 0.00274226 | 0.33523 | 1.15209 | VAP- up vs VAP+ |
| 40569_at | MZF1 | myeloid zinc finger 1 | 0.00278716 | 0.33523 | 1.15179 | VAP- up vs VAP+ |
| 218369_s_at | EXOSC1 | exosome component 1 | 0.00282878 | 0.33523 | -1.08353 | VAP- down vs VAP+ |
| 204732_s_at | TRIM23 | tripartite motif-containing 23 | 0.00287206 | 0.33523 | -1.11105 | VAP- down vs VAP+ |
| 203970_s_at | PEX3 | peroxisomal biogenesis factor 3 | 0.00293613 | 0.33523 | 1.16483 | VAP- up vs VAP+ |
| 201356_at | SF3A1 | splicing factor 3a, subunit 1, 120kDa | 0.00301894 | 0.33523 | 1.30935 | VAP- up vs VAP+ |
| 209333_at | ULK1 | unc-51-like kinase 1 (C. elegans) | 0.00305889 | 0.33523 | -1.32302 | VAP- down vs VAP+ |
| 202794_at | INPP1 | inositol polyphosphate-1-phosphatase | 0.00308359 | 0.33523 | 1.57782 | VAP- up vs VAP+ |
| 205538_at | CORO2A | coronin, actin binding protein, 2A | 0.00308738 | 0.33523 | -1.11575 | VAP- down vs VAP+ |
| 200679_x_at | HMGB1 | high-mobility group box 1 | 0.00310034 | 0.33523 | -1.2493 | VAP- down vs VAP+ |
| 208626_s_at | VAT1 | vesicle amine transport protein 1 homolog (T. californica) | 0.00314073 | 0.33523 | -1.13216 | VAP- down vs VAP+ |
| 207463_x_at | PRSS3 | protease, serine, 3 | 0.00314996 | 0.33523 | 1.18783 | VAP- up vs VAP+ |
| 204762_s_at | GNAO1 | guanine nucleotide binding protein (G protein), alpha activating activity polype | 0.00315751 | 0.33523 | -1.24865 | VAP- down vs VAP+ |
| 203919_at | TCEA2 | transcription elongation factor A (SII), 2 | 0.00321768 | 0.336595 | 1.11116 | VAP- up vs VAP+ |
| 209803_s_at | PHLDA2 | pleckstrin homology-like domain, family A, member 2 | 0.00334193 | 0.340401 | 2.09961 | VAP- up vs VAP+ |
| 203021_at | SLPI | secretory leukocyte peptidase inhibitor | 0.00334978 | 0.340401 | 1.85444 | VAP- up vs VAP+ |
| 220993_s_at | GPR63 | G protein-coupled receptor 63 | 0.00369497 | 0.362625 | -1.1631 | VAP- down vs VAP+ |
| 200825_s_at | HYOU1 | hypoxia up-regulated 1 | 0.00370422 | 0.362625 | 1.33906 | VAP- up vs VAP+ |
| 206964_at | NAT8B | N-acetyltransferase 8B (GCN5-related, putative, gene/pseudogene) | 0.00372141 | 0.362625 | 1.20225 | VAP- up vs VAP+ |
| 219788_at | PILRA | paired immunoglobin-like type 2 receptor alpha | 0.00382976 | 0.36453 | 1.35888 | VAP- up vs VAP+ |
| 204083_s_at | TPM2 | tropomyosin 2 (beta) | 0.00384345 | 0.36453 | 1.097 | VAP- up vs VAP+ |
| 204688_at | SGCE | sarcoglycan, epsilon | 0.00420181 | 0.379901 | 1.08385 | VAP- up vs VAP+ |
| 207339_s_at | LTB | lymphotoxin beta (TNF superfamily, member 3) | 0.00428055 | 0.379901 | 1.43049 | VAP- up vs VAP+ |
| 210191_s_at | PHTF1 | putative homeodomain transcription factor 1 | 0.00435406 | 0.379901 | -1.15718 | VAP- down vs VAP+ |
| 204603_at | EXO1 | exonuclease 1 | 0.00454184 | 0.379901 | -1.2057 | VAP- down vs VAP+ |
| 205013_s_at | ADORA2A /// CYTSA | adenosine A2a receptor /// cytospin A | 0.0045939 | 0.379901 | 1.28887 | VAP- up vs VAP+ |
| 202969_at | DYRK2 | dual-specificity tyrosine-(Y)-phosphorylation regulated kinase 2 | 0.0046533 | 0.379901 | 1.14103 | VAP- up vs VAP+ |
| 208612_at | PDIA3 | protein disulfide isomerase family A, member 3 | 0.00473512 | 0.379901 | 1.44546 | VAP- up vs VAP+ |
| 208516_at | MTNR1B | melatonin receptor 1B | 0.00483318 | 0.379901 | 1.15487 | VAP- up vs VAP+ |
| 215416_s_at | STOML2 | stomatin (EPB72)-like 2 | 0.00484308 | 0.379901 | 1.2458 | VAP- up vs VAP+ |
| 206115_at | EGR3 | early growth response 3 | 0.0048995 | 0.379901 | 1.5964 | VAP- up vs VAP+ |
| 203924_at | GSTA1 | glutathione S-transferase alpha 1 | 0.00491204 | 0.379901 | 1.09396 | VAP- up vs VAP+ |
| 221032_s_at | TMPRSS5 | transmembrane protease, serine 5 | 0.00494818 | 0.379901 | 1.20962 | VAP- up vs VAP+ |
| 200053_at | SPAG7 | sperm associated antigen 7 | 0.00501177 | 0.379901 | 1.24654 | VAP- up vs VAP+ |
| 37020_at | CRP | C-reactive protein, pentraxin-related | 0.00507144 | 0.379901 | -1.09271 | VAP- down vs VAP+ |
| 202615_at | GNAQ | Guanine nucleotide binding protein (G protein), q polypeptide | 0.00511001 | 0.379901 | -1.49353 | VAP- down vs VAP+ |
| 203943_at | KIF3B | kinesin family member 3B | 0.00515871 | 0.379901 | 1.67808 | VAP- up vs VAP+ |
| 212894_at | SUPV3L1 | suppressor of var1, 3-like 1 (S. cerevisiae) | 0.00516016 | 0.379901 | 1.15671 | VAP- up vs VAP+ |
| 207536_s_at | TNFRSF9 | tumor necrosis factor receptor superfamily, member 9 | 0.00523421 | 0.379901 | 1.43783 | VAP- up vs VAP+ |
| 207279_s_at | NEBL | nebulette | 0.00524612 | 0.379901 | 1.19452 | VAP- up vs VAP+ |
| 201095_at | DAP | death-associated protein | 0.00524776 | 0.379901 | 1.36075 | VAP- up vs VAP+ |
| 201042_at | TGM2 | transglutaminase 2 (C polypeptide, protein-glutamine-gamma-glutamyltransferase) | 0.00526892 | 0.379901 | 1.29651 | VAP- up vs VAP+ |
| 201707_at | PEX19 | peroxisomal biogenesis factor 19 | 0.00538042 | 0.379901 | -1.14766 | VAP- down vs VAP+ |
| 204850_s_at | DCX | doublecortin | 0.00542146 | 0.379901 | 1.10697 | VAP- up vs VAP+ |
| 220051_at | PRSS21 | protease, serine, 21 (testisin) | 0.00547759 | 0.379901 | -1.13346 | VAP- down vs VAP+ |
| 205723_at | CNTFR | ciliary neurotrophic factor receptor | 0.00548271 | 0.379901 | 1.36127 | VAP- up vs VAP+ |
| 210042_s_at | CTSZ | cathepsin Z | 0.00553966 | 0.379901 | 1.81865 | VAP- up vs VAP+ |
| 218120_s_at | HMOX2 | heme oxygenase (decycling) 2 | 0.00557391 | 0.379901 | 1.24748 | VAP- up vs VAP+ |
| 213263_s_at | PCBP2 | poly(rC) binding protein 2 | 0.00563532 | 0.379901 | -1.19171 | VAP- down vs VAP+ |
| 207226_at | HIST1H2BN | histone cluster 1, H2bn | 0.00577122 | 0.379901 | 1.15192 | VAP- up vs VAP+ |
| 207329_at | MMP8 | matrix metallopeptidase 8 (neutrophil collagenase) | 0.00577581 | 0.379901 | -3.65899 | VAP- down vs VAP+ |
| 204787_at | VSIG4 | V-set and immunoglobulin domain containing 4 | 0.00583709 | 0.379901 | -1.12813 | VAP- down vs VAP+ |
| 210037_s_at | NOS2 | nitric oxide synthase 2, inducible | 0.00588585 | 0.379901 | 1.16712 | VAP- up vs VAP+ |
| 212360_at | AMPD2 | adenosine monophosphate deaminase 2 | 0.0059183 | 0.379901 | 1.30281 | VAP- up vs VAP+ |
| 34408_at | RTN2 | reticulon 2 | 0.00594066 | 0.379901 | 1.49011 | VAP- up vs VAP+ |
| 205452_at | PIGB | phosphatidylinositol glycan anchor biosynthesis, class B | 0.00597004 | 0.379901 | -1.39739 | VAP- down vs VAP+ |
| 210052_s_at | TPX2 | TPX2, microtubule-associated, homolog (Xenopus laevis) | 0.00609911 | 0.379901 | -1.26578 | VAP- down vs VAP+ |
| 217767_at | C3 | complement component 3 | 0.00613357 | 0.379901 | 1.52128 | VAP- up vs VAP+ |
| 217722_s_at | NGRN | neugrin, neurite outgrowth associated | 0.00613981 | 0.379901 | 1.30483 | VAP- up vs VAP+ |
| 202764_at | STIM1 | stromal interaction molecule 1 | 0.00617116 | 0.379901 | -1.24704 | VAP- down vs VAP+ |
| 200968_s_at | PPIB | peptidylprolyl isomerase B (cyclophilin B) | 0.00623622 | 0.379901 | 1.52437 | VAP- up vs VAP+ |
| 203050_at | TP53BP1 | tumor protein p53 binding protein 1 | 0.00628067 | 0.379901 | 1.24215 | VAP- up vs VAP+ |
| 220746_s_at | UIMC1 | ubiquitin interaction motif containing 1 | 0.00629892 | 0.379901 | -1.19879 | VAP- down vs VAP+ |
| 207080_s_at | PYY | peptide YY | 0.00631876 | 0.379901 | 1.09884 | VAP- up vs VAP+ |
| 201000_at | AARS | alanyl-tRNA synthetase | 0.00643056 | 0.379901 | 1.22408 | VAP- up vs VAP+ |
| 206523_at | CYTH3 | cytohesin 3 | 0.00643215 | 0.379901 | -1.15039 | VAP- down vs VAP+ |
| 217923_at | PEF1 | penta-EF-hand domain containing 1 | 0.00646223 | 0.379901 | 1.32804 | VAP- up vs VAP+ |
| 213553_x_at | APOC1 | apolipoprotein C-I | 0.00657448 | 0.382013 | 1.13294 | VAP- up vs VAP+ |
| 201037_at | PFKP | phosphofructokinase, platelet | 0.00671926 | 0.382013 | 1.26989 | VAP- up vs VAP+ |
| 221106_at | SLC22A17 | solute carrier family 22, member 17 | 0.00675131 | 0.382013 | 1.11718 | VAP- up vs VAP+ |
| 200877_at | CCT4 | chaperonin containing TCP1, subunit 4 (delta) | 0.00676027 | 0.382013 | 1.34186 | VAP- up vs VAP+ |
| 203043_at | ZBED1 | zinc finger, BED-type containing 1 | 0.00676668 | 0.382013 | -1.22605 | VAP- down vs VAP+ |
| 206762_at | KCNA5 | potassium voltage-gated channel, shaker-related subfamily, member 5 | 0.00694571 | 0.382789 | 1.14015 | VAP- up vs VAP+ |
| 203325_s_at | COL5A1 | collagen, type V, alpha 1 | 0.00702987 | 0.382789 | -1.16163 | VAP- down vs VAP+ |
| 206097_at | SLC22A18AS | solute carrier family 22 (organic cation transporter), member 18 antisense | 0.00703275 | 0.382789 | 1.17605 | VAP- up vs VAP+ |
| 219205_at | SRR | serine racemase | 0.00704019 | 0.382789 | -1.15163 | VAP- down vs VAP+ |
| 221338_at | LY6G6E | lymphocyte antigen 6 complex, locus G6E | 0.00706757 | 0.382789 | 1.08844 | VAP- up vs VAP+ |
| 206177_s_at | ARG1 | arginase, liver | 0.00723149 | 0.382789 | -2.80647 | VAP- down vs VAP+ |
| 207413_s_at | SCN5A | sodium channel, voltage-gated, type V, alpha subunit | 0.00731117 | 0.382789 | -1.17963 | VAP- down vs VAP+ |
| 209879_at | SELPLG | selectin P ligand | 0.00732181 | 0.382789 | -1.64079 | VAP- down vs VAP+ |
| 220365_at | ALLC | allantoicase | 0.00774431 | 0.382789 | -1.19442 | VAP- down vs VAP+ |
| 205741_s_at | DTNA | dystrobrevin, alpha | 0.00779284 | 0.382789 | 1.17699 | VAP- up vs VAP+ |
| 221133_s_at | CLDN18 | claudin 18 | 0.00788364 | 0.382789 | -1.15629 | VAP- down vs VAP+ |
| 210117_at | SPAG1 | sperm associated antigen 1 | 0.00801429 | 0.382789 | -1.28715 | VAP- down vs VAP+ |
| 207595_s_at | BMP1 | bone morphogenetic protein 1 | 0.00802932 | 0.382789 | 1.12418 | VAP- up vs VAP+ |
| 212048_s_at | YARS | tyrosyl-tRNA synthetase | 0.00803085 | 0.382789 | 1.19122 | VAP- up vs VAP+ |
| 205979_at | SCGB2A1 | secretoglobin, family 2A, member 1 | 0.00804031 | 0.382789 | 1.1023 | VAP- up vs VAP+ |
| 207906_at | IL3 | interleukin 3 (colony-stimulating factor, multiple) | 0.00804678 | 0.382789 | -1.11401 | VAP- down vs VAP+ |
| 208895_s_at | DDX18 | DEAD (Asp-Glu-Ala-Asp) box polypeptide 18 | 0.00818276 | 0.382789 | 1.4358 | VAP- up vs VAP+ |
| 204497_at | ADCY9 | adenylate cyclase 9 | 0.00826462 | 0.382789 | 1.09349 | VAP- up vs VAP+ |
| 202224_at | CRK | v-crk sarcoma virus CT10 oncogene homolog (avian) | 0.00833588 | 0.382789 | 1.56438 | VAP- up vs VAP+ |
| 209433_s_at | PPAT | phosphoribosyl pyrophosphate amidotransferase | 0.0083892 | 0.382789 | -1.16314 | VAP- down vs VAP+ |
| 206430_at | CDX1 | caudal type homeobox 1 | 0.00839108 | 0.382789 | 1.0976 | VAP- up vs VAP+ |
| 202582_s_at | RANBP9 | RAN binding protein 9 | 0.00849744 | 0.382789 | -1.48717 | VAP- down vs VAP+ |
| 211841_s_at | TNFRSF25 | tumor necrosis factor receptor superfamily, member 25 | 0.0085389 | 0.382789 | 1.30266 | VAP- up vs VAP+ |
| 204070_at | RARRES3 | retinoic acid receptor responder (tazarotene induced) 3 | 0.00853957 | 0.382789 | 1.389 | VAP- up vs VAP+ |
| 1494_f_at | CYP2A6 | cytochrome P450, family 2, subfamily A, polypeptide 6 | 0.00859897 | 0.382789 | 1.10394 | VAP- up vs VAP+ |
| 218523_at | LHPP | phospholysine phosphohistidine inorganic pyrophosphate phosphatase | 0.00866522 | 0.382789 | -1.19114 | VAP- down vs VAP+ |
| 201480_s_at | SUPT5H | suppressor of Ty 5 homolog (S. cerevisiae) | 0.00866598 | 0.382789 | 1.20477 | VAP- up vs VAP+ |
| 219452_at | DPEP2 | dipeptidase 2 | 0.00870026 | 0.382789 | 1.50924 | VAP- up vs VAP+ |
| 209892_at | FUT4 | fucosyltransferase 4 (alpha (1,3) fucosyltransferase, myeloid-specific) | 0.00871927 | 0.382789 | -1.39465 | VAP- down vs VAP+ |
| 203284_s_at | HS2ST1 | heparan sulfate 2-O-sulfotransferase 1 | 0.00872675 | 0.382789 | -1.25599 | VAP- down vs VAP+ |
| 205579_at | HRH1 | histamine receptor H1 | 0.00886924 | 0.382789 | 1.35863 | VAP- up vs VAP+ |
| 204588_s_at | SLC7A7 | solute carrier family 7 (cationic amino acid transporter, y+ system), member 7 | 0.00890695 | 0.382789 | 1.47434 | VAP- up vs VAP+ |
| 205445_at | PRL | prolactin | 0.00890769 | 0.382789 | -1.28587 | VAP- down vs VAP+ |
| 207409_at | LECT2 | leukocyte cell-derived chemotaxin 2 | 0.00893778 | 0.382789 | 1.12419 | VAP- up vs VAP+ |
| 206995_x_at | SCARF1 | scavenger receptor class F, member 1 | 0.00895557 | 0.382789 | 1.52197 | VAP- up vs VAP+ |
| 53912_at | SNX11 | sorting nexin 11 | 0.00920243 | 0.382789 | -1.10376 | VAP- down vs VAP+ |
| 207636_at | SERPINI2 | serpin peptidase inhibitor, clade I (pancpin), member 2 | 0.00927243 | 0.382789 | -1.19369 | VAP- down vs VAP+ |
| 200982_s_at | ANXA6 | annexin A6 | 0.0094294 | 0.382789 | -1.29356 | VAP- down vs VAP+ |
| 221254_s_at | PITPNM3 | PITPNM family member 3 | 0.00944981 | 0.382789 | 1.11467 | VAP- up vs VAP+ |
| 207182_at | GABRA6 | gamma-aminobutyric acid (GABA) A receptor, alpha 6 | 0.00948458 | 0.382789 | -1.16211 | VAP- down vs VAP+ |
| 206105_at | AFF2 | AF4/FMR2 family, member 2 | 0.00959795 | 0.382789 | 1.20455 | VAP- up vs VAP+ |
| 221394_at | TAAR2 | trace amine associated receptor 2 | 0.00963011 | 0.382789 | 1.1922 | VAP- up vs VAP+ |
| 201362_at | IVNS1ABP | influenza virus NS1A binding protein | 0.00973133 | 0.382789 | 1.41439 | VAP- up vs VAP+ |
| 202283_at | SERPINF1 | serpin peptidase inhibitor, clade F (alpha-2 antiplasmin, pigment epithelium der | 0.00975277 | 0.382789 | 1.13874 | VAP- up vs VAP+ |
| 205774_at | F12 | coagulation factor XII (Hageman factor) | 0.0097836 | 0.382789 | 1.11463 | VAP- up vs VAP+ |
| 218718_at | PDGFC | platelet derived growth factor C | 0.00978698 | 0.382789 | -1.71994 | VAP- down vs VAP+ |
| 211474_s_at | SERPINB6 | serpin peptidase inhibitor, clade B (ovalbumin), member 6 | 0.00986908 | 0.382789 | 1.32393 | VAP- up vs VAP+ |
| 202668_at | EFNB2 | ephrin-B2 | 0.0100384 | 0.382789 | -1.16293 | VAP- down vs VAP+ |
| 204794_at | DUSP2 | dual specificity phosphatase 2 | 0.0100931 | 0.382789 | 1.94693 | VAP- up vs VAP+ |
| 207300_s_at | F7 | coagulation factor VII (serum prothrombin conversion accelerator) | 0.0101545 | 0.382789 | 1.17117 | VAP- up vs VAP+ |
| 215999_at | CDRT1 | CMT1A duplicated region transcript 1 | 0.0101996 | 0.382789 | 1.14192 | VAP- up vs VAP+ |
| 209043_at | PAPSS1 | 3'-phosphoadenosine 5'-phosphosulfate synthase 1 | 0.0102232 | 0.382789 | -1.53882 | VAP- down vs VAP+ |
| 220065_at | TNMD | tenomodulin | 0.0102516 | 0.382789 | -1.18192 | VAP- down vs VAP+ |
| 203729_at | EMP3 | epithelial membrane protein 3 | 0.0103707 | 0.382789 | 1.2493 | VAP- up vs VAP+ |
| 220778_x_at | SEMA6B | sema domain, transmembrane domain (TM), and cytoplasmic domain, (semaphorin) 6B | 0.0104505 | 0.382789 | 1.2156 | VAP- up vs VAP+ |
| 202284_s_at | CDKN1A | cyclin-dependent kinase inhibitor 1A (p21, Cip1) | 0.0106293 | 0.382789 | 1.52469 | VAP- up vs VAP+ |
| 201489_at | PPIF | peptidylprolyl isomerase F | 0.0107226 | 0.382789 | 1.8305 | VAP- up vs VAP+ |
| 210246_s_at | ABCC8 | ATP-binding cassette, sub-family C (CFTR/MRP), member 8 | 0.0107441 | 0.382789 | 1.17642 | VAP- up vs VAP+ |
| 210116_at | SH2D1A | SH2 domain containing 1A | 0.0107502 | 0.382789 | 1.28879 | VAP- up vs VAP+ |
| 209949_at | NCF2 | neutrophil cytosolic factor 2 | 0.0108036 | 0.382789 | 1.33085 | VAP- up vs VAP+ |
| 208768_x_at | RPL22 | ribosomal protein L22 | 0.0109563 | 0.382789 | 1.4224 | VAP- up vs VAP+ |
| 200593_s_at | HNRNPU | heterogeneous nuclear ribonucleoprotein U (scaffold attachment factor A) | 0.0109893 | 0.382789 | 1.23171 | VAP- up vs VAP+ |
| 208634_s_at | MACF1 | microtubule-actin crosslinking factor 1 | 0.0110065 | 0.382789 | -1.23399 | VAP- down vs VAP+ |
| 211284_s_at | GRN | granulin | 0.0110065 | 0.382789 | -1.33761 | VAP- down vs VAP+ |
| 221421_s_at | ADAMTS12 | ADAM metallopeptidase with thrombospondin type 1 motif, 12 | 0.0110251 | 0.382789 | 1.16558 | VAP- up vs VAP+ |
| 215983_s_at | UBXN8 | UBX domain protein 8 | 0.0110447 | 0.382789 | 1.10649 | VAP- up vs VAP+ |
| 201192_s_at | PITPNA | phosphatidylinositol transfer protein, alpha | 0.0110524 | 0.382789 | 1.46851 | VAP- up vs VAP+ |
| 201511_at | AAMP | angio-associated, migratory cell protein | 0.0110843 | 0.382789 | 1.19595 | VAP- up vs VAP+ |
| 200990_at | TRIM28 | tripartite motif-containing 28 | 0.0110945 | 0.382789 | 1.43647 | VAP- up vs VAP+ |
| 206517_at | CDH16 | cadherin 16, KSP-cadherin | 0.0112195 | 0.382789 | 1.21691 | VAP- up vs VAP+ |
| 211980_at | COL4A1 | collagen, type IV, alpha 1 | 0.011263 | 0.382789 | 1.08595 | VAP- up vs VAP+ |
| 205317_s_at | SLC15A2 | solute carrier family 15 (H+/peptide transporter), member 2 | 0.0114368 | 0.382789 | 1.14745 | VAP- up vs VAP+ |
| 204881_s_at | UGCG | UDP-glucose ceramide glucosyltransferase | 0.0115095 | 0.382789 | -1.8001 | VAP- down vs VAP+ |
| 213467_at | RND2 | Rho family GTPase 2 | 0.0115391 | 0.382789 | -1.22514 | VAP- down vs VAP+ |
| 219398_at | CIDEC | cell death-inducing DFFA-like effector c | 0.0115996 | 0.382789 | 1.13236 | VAP- up vs VAP+ |
| 201236_s_at | BTG2 | BTG family, member 2 | 0.0116278 | 0.382789 | 1.5137 | VAP- up vs VAP+ |
| 219568_x_at | SOX18 | SRY (sex determining region Y)-box 18 | 0.0116318 | 0.382789 | 1.10508 | VAP- up vs VAP+ |
| 204544_at | HPS5 | Hermansky-Pudlak syndrome 5 | 0.0116434 | 0.382789 | 1.34788 | VAP- up vs VAP+ |
| 201284_s_at | APEH | N-acylaminoacyl-peptide hydrolase | 0.0116466 | 0.382789 | 1.10965 | VAP- up vs VAP+ |
| 209587_at | PITX1 | paired-like homeodomain 1 | 0.0116753 | 0.382789 | 1.0722 | VAP- up vs VAP+ |
| 208046_at | HIST1H4A | histone cluster 1, H4a | 0.0116774 | 0.382789 | 1.13704 | VAP- up vs VAP+ |
| 221216_s_at | SCMH1 | sex comb on midleg homolog 1 (Drosophila) | 0.0117929 | 0.382789 | 1.1188 | VAP- up vs VAP+ |
| 210270_at | RGS6 | regulator of G-protein signaling 6 | 0.0119061 | 0.382789 | -1.11805 | VAP- down vs VAP+ |
| 203927_at | NFKBIE | nuclear factor of kappa light polypeptide gene enhancer in B-cells inhibitor, ep | 0.0119443 | 0.382789 | 1.44749 | VAP- up vs VAP+ |
| 201681_s_at | DLG5 | discs, large homolog 5 (Drosophila) | 0.0120351 | 0.382789 | -1.10124 | VAP- down vs VAP+ |
| 220759_at | EDDM3B | epididymal protein 3B | 0.0121115 | 0.382789 | -1.20731 | VAP- down vs VAP+ |
| 217004_s_at | MCF2 | MCF.2 cell line derived transforming sequence | 0.0121138 | 0.382789 | 1.11547 | VAP- up vs VAP+ |
| 208880_s_at | PRPF6 | PRP6 pre-mRNA processing factor 6 homolog (S. cerevisiae) | 0.0121595 | 0.382789 | 1.19502 | VAP- up vs VAP+ |
| 201994_at | MORF4L2 | mortality factor 4 like 2 | 0.012166 | 0.382789 | 1.65024 | VAP- up vs VAP+ |
| 214062_x_at | NFKBIB | nuclear factor of kappa light polypeptide gene enhancer in B-cells inhibitor, be | 0.0122052 | 0.382789 | 1.28675 | VAP- up vs VAP+ |
| 206074_s_at | HMGA1 | high mobility group AT-hook 1 | 0.0123269 | 0.382789 | 1.46473 | VAP- up vs VAP+ |
| 201884_at | CEACAM5 | carcinoembryonic antigen-related cell adhesion molecule 5 | 0.0124512 | 0.382789 | 1.15976 | VAP- up vs VAP+ |
| 220403_s_at | TP53AIP1 | tumor protein p53 regulated apoptosis inducing protein 1 | 0.0124785 | 0.382789 | -1.11722 | VAP- down vs VAP+ |
| 208662_s_at | TTC3 | tetratricopeptide repeat domain 3 | 0.0125442 | 0.382789 | 1.16458 | VAP- up vs VAP+ |
| 203391_at | FKBP2 | FK506 binding protein 2, 13kDa | 0.0125458 | 0.382789 | 1.28598 | VAP- up vs VAP+ |
| 217749_at | COPG | coatomer protein complex, subunit gamma | 0.0125622 | 0.382789 | 1.15166 | VAP- up vs VAP+ |
| 220528_at | VNN3 | vanin 3 | 0.0127496 | 0.382789 | 1.48699 | VAP- up vs VAP+ |
| 205733_at | BLM | Bloom syndrome, RecQ helicase-like | 0.0127673 | 0.382789 | -1.29557 | VAP- down vs VAP+ |
| 213136_at | PTPN2 | protein tyrosine phosphatase, non-receptor type 2 | 0.0128037 | 0.382789 | -1.20006 | VAP- down vs VAP+ |
| 203228_at | PAFAH1B3 | platelet-activating factor acetylhydrolase 1b, catalytic subunit 3 (29kDa) | 0.012865 | 0.382789 | 1.23976 | VAP- up vs VAP+ |
| 200901_s_at | M6PR | mannose-6-phosphate receptor (cation dependent) | 0.012867 | 0.382789 | 1.33292 | VAP- up vs VAP+ |
| 206380_s_at | CFP | complement factor properdin | 0.0128856 | 0.382789 | 1.30111 | VAP- up vs VAP+ |
| 219350_s_at | DIABLO | diablo homolog (Drosophila) | 0.0129496 | 0.382789 | 1.20269 | VAP- up vs VAP+ |
| 205042_at | GNE | glucosamine (UDP-N-acetyl)-2-epimerase/N-acetylmannosamine kinase | 0.0129498 | 0.382789 | 1.11634 | VAP- up vs VAP+ |
| 202655_at | MANF | mesencephalic astrocyte-derived neurotrophic factor | 0.0129691 | 0.382789 | 1.22092 | VAP- up vs VAP+ |
| 201193_at | IDH1 | isocitrate dehydrogenase 1 (NADP+), soluble | 0.0129851 | 0.382789 | -1.2789 | VAP- down vs VAP+ |
| 206278_at | PTAFR | platelet-activating factor receptor | 0.0129874 | 0.382789 | 1.26309 | VAP- up vs VAP+ |
| 202717_s_at | CDC16 | cell division cycle 16 homolog (S. cerevisiae) | 0.0129875 | 0.382789 | 1.19064 | VAP- up vs VAP+ |
| 203262_s_at | FAM50A | family with sequence similarity 50, member A | 0.0130181 | 0.382789 | 1.36862 | VAP- up vs VAP+ |
| 202419_at | KDSR | 3-ketodihydrosphingosine reductase | 0.0130216 | 0.382789 | 1.17796 | VAP- up vs VAP+ |
| 206233_at | B4GALT6 | UDP-Gal:betaGlcNAc beta 1,4- galactosyltransferase, polypeptide 6 | 0.0130336 | 0.382789 | -1.14523 | VAP- down vs VAP+ |
| 206697_s_at | HP | haptoglobin | 0.0130953 | 0.382789 | -2.66005 | VAP- down vs VAP+ |
| 209111_at | RNF5 | ring finger protein 5 | 0.0131382 | 0.382789 | -1.12422 | VAP- down vs VAP+ |
| 202042_at | HARS | histidyl-tRNA synthetase | 0.0131455 | 0.382789 | 1.2085 | VAP- up vs VAP+ |
| 205701_at | IPO8 | importin 8 | 0.0131815 | 0.382789 | 1.15834 | VAP- up vs VAP+ |
| 204534_at | VTN | vitronectin | 0.013208 | 0.382789 | 1.20185 | VAP- up vs VAP+ |
| 208756_at | EIF3I | eukaryotic translation initiation factor 3, subunit I | 0.0132609 | 0.382789 | 1.21245 | VAP- up vs VAP+ |
| 207026_s_at | ATP2B3 | ATPase, Ca++ transporting, plasma membrane 3 | 0.0132783 | 0.382789 | 1.17152 | VAP- up vs VAP+ |
| 205216_s_at | APOH | apolipoprotein H (beta-2-glycoprotein I) | 0.0133455 | 0.382789 | 1.11388 | VAP- up vs VAP+ |
| 207847_s_at | MUC1 | mucin 1, cell surface associated | 0.0133788 | 0.382789 | -1.15234 | VAP- down vs VAP+ |
| 202758_s_at | RFXANK | regulatory factor X-associated ankyrin-containing protein | 0.0134079 | 0.382789 | 1.13286 | VAP- up vs VAP+ |
| 209432_s_at | CREB3 | cAMP responsive element binding protein 3 | 0.013435 | 0.382789 | 1.23547 | VAP- up vs VAP+ |
| 200599_s_at | HSP90B1 | heat shock protein 90kDa beta (Grp94), member 1 | 0.0134368 | 0.382789 | 1.46668 | VAP- up vs VAP+ |
| 203965_at | USP20 | ubiquitin specific peptidase 20 | 0.0134818 | 0.382789 | 1.21163 | VAP- up vs VAP+ |
| 211225_at | FUT5 | fucosyltransferase 5 (alpha (1,3) fucosyltransferase) | 0.0135726 | 0.382789 | 1.15169 | VAP- up vs VAP+ |
| 213086_s_at | CSNK1A1 | casein kinase 1, alpha 1 | 0.0136461 | 0.382789 | 1.32652 | VAP- up vs VAP+ |
| 200002_at | RPL35 | ribosomal protein L35 | 0.0136936 | 0.382789 | 1.57659 | VAP- up vs VAP+ |
| 205621_at | ALKBH1 | alkB, alkylation repair homolog 1 (E. coli) | 0.013715 | 0.382789 | -1.16627 | VAP- down vs VAP+ |
| 203229_s_at | CLK2 | CDC-like kinase 2 | 0.0137223 | 0.382789 | 1.24025 | VAP- up vs VAP+ |
| 40560_at | TBX2 | T-box 2 | 0.0138737 | 0.382879 | 1.0836 | VAP- up vs VAP+ |
| 210305_at | PDE4DIP | phosphodiesterase 4D interacting protein | 0.013886 | 0.382879 | 1.24076 | VAP- up vs VAP+ |
| 210445_at | FABP6 | fatty acid binding protein 6, ileal | 0.0139142 | 0.382879 | 1.13554 | VAP- up vs VAP+ |
| 206618_at | IL18R1 | interleukin 18 receptor 1 | 0.0139408 | 0.382879 | -2.22859 | VAP- down vs VAP+ |
| 208551_at | HIST1H4G | histone cluster 1, H4g | 0.0140816 | 0.385258 | 1.13212 | VAP- up vs VAP+ |
| 206828_at | TXK | TXK tyrosine kinase | 0.0141794 | 0.385795 | 1.23879 | VAP- up vs VAP+ |
| 218030_at | GIT1 | G protein-coupled receptor kinase interacting ArfGAP 1 | 0.0142757 | 0.385795 | 1.17813 | VAP- up vs VAP+ |
| 207635_s_at | KCNH1 | potassium voltage-gated channel, subfamily H (eag-related), member 1 | 0.0143553 | 0.385795 | -1.1382 | VAP- down vs VAP+ |
| 208881_x_at | IDI1 | isopentenyl-diphosphate delta isomerase 1 | 0.0144317 | 0.385795 | -1.52432 | VAP- down vs VAP+ |
| 205525_at | CALD1 | caldesmon 1 | 0.014457 | 0.385795 | -1.14458 | VAP- down vs VAP+ |
| 204313_s_at | CREB1 | cAMP responsive element binding protein 1 | 0.014466 | 0.385795 | 1.33974 | VAP- up vs VAP+ |
| 212517_at | ATRN | attractin | 0.0145137 | 0.385795 | -1.18123 | VAP- down vs VAP+ |
| 207144_s_at | CITED1 | Cbp/p300-interacting transactivator, with Glu/Asp-rich carboxy-terminal domain, | 0.0145351 | 0.385795 | 1.13607 | VAP- up vs VAP+ |
| 220604_x_at | FTCD | formiminotransferase cyclodeaminase | 0.0146371 | 0.386185 | 1.21867 | VAP- up vs VAP+ |
| 204893_s_at | ZFYVE9 | zinc finger, FYVE domain containing 9 | 0.0146584 | 0.386185 | 1.14396 | VAP- up vs VAP+ |
| 201069_at | MMP2 | matrix metallopeptidase 2 (gelatinase A, 72kDa gelatinase, 72kDa type IV collage | 0.0148663 | 0.390185 | 1.10031 | VAP- up vs VAP+ |
| 202392_s_at | PISD | phosphatidylserine decarboxylase | 0.0149199 | 0.390185 | 1.33109 | VAP- up vs VAP+ |
| 210202_s_at | BIN1 | bridging integrator 1 | 0.0150455 | 0.391685 | 1.23816 | VAP- up vs VAP+ |
| 209765_at | ADAM19 | ADAM metallopeptidase domain 19 (meltrin beta) | 0.0150874 | 0.391685 | -1.33126 | VAP- down vs VAP+ |
| 40687_at | GJA4 | gap junction protein, alpha 4, 37kDa | 0.0153222 | 0.392551 | 1.08056 | VAP- up vs VAP+ |
| 204993_at | GNAZ | guanine nucleotide binding protein (G protein), alpha z polypeptide | 0.0153303 | 0.392551 | 1.22566 | VAP- up vs VAP+ |
| 201397_at | PHGDH | phosphoglycerate dehydrogenase | 0.0153962 | 0.392551 | 1.15121 | VAP- up vs VAP+ |
| 209012_at | TRIO | triple functional domain (PTPRF interacting) | 0.0154083 | 0.392551 | 1.09026 | VAP- up vs VAP+ |
| 200873_s_at | CCT8 | chaperonin containing TCP1, subunit 8 (theta) | 0.0154178 | 0.392551 | 1.4117 | VAP- up vs VAP+ |
| 204112_s_at | HNMT | histamine N-methyltransferase | 0.0157175 | 0.392551 | 1.30003 | VAP- up vs VAP+ |
| 201739_at | SGK1 | serum/glucocorticoid regulated kinase 1 | 0.015734 | 0.392551 | 1.80811 | VAP- up vs VAP+ |
| 203887_s_at | THBD | thrombomodulin | 0.0157347 | 0.392551 | 1.82949 | VAP- up vs VAP+ |
| 206368_at | CPLX2 | complexin 2 | 0.0158993 | 0.392551 | -1.14078 | VAP- down vs VAP+ |
| 204432_at | SOX12 | SRY (sex determining region Y)-box 12 | 0.0159357 | 0.392551 | 1.15239 | VAP- up vs VAP+ |
| 211169_s_at | PPP1R3A | protein phosphatase 1, regulatory (inhibitor) subunit 3A | 0.0159864 | 0.392551 | -1.07081 | VAP- down vs VAP+ |
| 203080_s_at | BAZ2B | bromodomain adjacent to zinc finger domain, 2B | 0.0160392 | 0.392551 | -1.31293 | VAP- down vs VAP+ |
| 206820_at | AGFG2 | ArfGAP with FG repeats 2 | 0.0160907 | 0.392551 | -1.15514 | VAP- down vs VAP+ |
| 200078_s_at | ATP6V0B | ATPase, H+ transporting, lysosomal 21kDa, V0 subunit b | 0.0160967 | 0.392551 | 1.33777 | VAP- up vs VAP+ |
| 204862_s_at | NME3 | non-metastatic cells 3, protein expressed in | 0.0162128 | 0.392551 | 1.16391 | VAP- up vs VAP+ |
| 209438_at | PHKA2 | phosphorylase kinase, alpha 2 (liver) | 0.0162335 | 0.392551 | -1.22118 | VAP- down vs VAP+ |
| 210028_s_at | ORC3L | origin recognition complex, subunit 3-like (yeast) | 0.0162423 | 0.392551 | 1.10916 | VAP- up vs VAP+ |
| 204683_at | ICAM2 | intercellular adhesion molecule 2 | 0.0162426 | 0.392551 | 1.34857 | VAP- up vs VAP+ |
| 207933_at | ZP2 | zona pellucida glycoprotein 2 (sperm receptor) | 0.016289 | 0.392551 | 1.2004 | VAP- up vs VAP+ |
| 206831_s_at | ARSD | arylsulfatase D | 0.0163064 | 0.392551 | 1.25416 | VAP- up vs VAP+ |
| 214401_at | PAX1 | paired box 1 | 0.0163633 | 0.392551 | 1.10356 | VAP- up vs VAP+ |
| 201730_s_at | TPR | translocated promoter region (to activated MET oncogene) | 0.0164121 | 0.392551 | -1.36182 | VAP- down vs VAP+ |
| 213330_s_at | STIP1 | stress-induced-phosphoprotein 1 | 0.0164644 | 0.392551 | 1.217 | VAP- up vs VAP+ |
| 206739_at | HOXC5 | homeobox C5 | 0.0164646 | 0.392551 | 1.15288 | VAP- up vs VAP+ |
| 200089_s_at | RPL4 | ribosomal protein L4 | 0.0165004 | 0.392551 | 1.53121 | VAP- up vs VAP+ |
| 206562_s_at | CSNK1A1 | casein kinase 1, alpha 1 | 0.0166063 | 0.392644 | 1.27204 | VAP- up vs VAP+ |
| 212642_s_at | HIVEP2 | human immunodeficiency virus type I enhancer binding protein 2 | 0.0166147 | 0.392644 | 1.25029 | VAP- up vs VAP+ |
| 201258_at | RPS16 | ribosomal protein S16 | 0.0167606 | 0.393947 | 1.47257 | VAP- up vs VAP+ |
| 204140_at | TPST1 | tyrosylprotein sulfotransferase 1 | 0.0169146 | 0.393947 | -1.36673 | VAP- down vs VAP+ |
| 203593_at | CD2AP | CD2-associated protein | 0.0169515 | 0.393947 | 1.10917 | VAP- up vs VAP+ |
| 207462_at | GLRA2 | glycine receptor, alpha 2 | 0.0169612 | 0.393947 | -1.27175 | VAP- down vs VAP+ |
| 221116_at | --- | --- | 0.0169798 | 0.393947 | 1.13351 | VAP- up vs VAP+ |
| 204951_at | RHOH | ras homolog gene family, member H | 0.0170327 | 0.393947 | -1.44243 | VAP- down vs VAP+ |
| 217849_s_at | CDC42BPB | CDC42 binding protein kinase beta (DMPK-like) | 0.0170575 | 0.393947 | 1.11144 | VAP- up vs VAP+ |
| 220066_at | NOD2 | nucleotide-binding oligomerization domain containing 2 | 0.0171232 | 0.394185 | 1.74271 | VAP- up vs VAP+ |
| 219762_s_at | RPL36 | ribosomal protein L36 | 0.0171824 | 0.394271 | 1.43624 | VAP- up vs VAP+ |
| 213551_x_at | PCGF2 | polycomb group ring finger 2 | 0.017362 | 0.396939 | 1.19249 | VAP- up vs VAP+ |
| 37950_at | PREP | prolyl endopeptidase | 0.0175195 | 0.396939 | 1.06453 | VAP- up vs VAP+ |
| 200054_at | ZNF259 | zinc finger protein 259 | 0.0175626 | 0.396939 | 1.19455 | VAP- up vs VAP+ |
| 207421_at | CA5A | carbonic anhydrase VA, mitochondrial | 0.0175638 | 0.396939 | 1.08833 | VAP- up vs VAP+ |
| 212255_s_at | ATP2C1 | ATPase, Ca++ transporting, type 2C, member 1 | 0.0177569 | 0.396939 | 1.10185 | VAP- up vs VAP+ |
| 218952_at | PCSK1N | proprotein convertase subtilisin/kexin type 1 inhibitor | 0.0177688 | 0.396939 | 1.14381 | VAP- up vs VAP+ |
| 210761_s_at | GRB7 | growth factor receptor-bound protein 7 | 0.0178436 | 0.396939 | 1.21492 | VAP- up vs VAP+ |
| 206871_at | ELANE | elastase, neutrophil expressed | 0.0179084 | 0.396939 | -1.68753 | VAP- down vs VAP+ |
| 205081_at | CRIP1 | cysteine-rich protein 1 (intestinal) | 0.0179362 | 0.396939 | 1.53172 | VAP- up vs VAP+ |
| 201851_at | SH3GL1 | SH3-domain GRB2-like 1 | 0.018006 | 0.396939 | 1.26492 | VAP- up vs VAP+ |
| 212321_at | SGPL1 | sphingosine-1-phosphate lyase 1 | 0.0180649 | 0.396939 | 1.09029 | VAP- up vs VAP+ |
| 202575_at | CRABP2 | cellular retinoic acid binding protein 2 | 0.0180707 | 0.396939 | 1.16534 | VAP- up vs VAP+ |
| 202237_at | NNMT | nicotinamide N-methyltransferase | 0.0182009 | 0.396939 | 1.16553 | VAP- up vs VAP+ |
| 37408_at | MRC2 | mannose receptor, C type 2 | 0.0182875 | 0.396939 | 1.1174 | VAP- up vs VAP+ |
| 218032_at | SNN | stannin | 0.018297 | 0.396939 | 1.38936 | VAP- up vs VAP+ |
| 218145_at | TRIB3 | tribbles homolog 3 (Drosophila) | 0.0183328 | 0.396939 | 1.39966 | VAP- up vs VAP+ |
| 211570_s_at | RAPSN | receptor-associated protein of the synapse | 0.018337 | 0.396939 | 1.14565 | VAP- up vs VAP+ |
| 208584_at | SNCG | synuclein, gamma (breast cancer-specific protein 1) | 0.0183764 | 0.396939 | 1.24778 | VAP- up vs VAP+ |
| 203766_s_at | LMOD1 | leiomodin 1 (smooth muscle) | 0.0183812 | 0.396939 | 1.15505 | VAP- up vs VAP+ |
| 205182_s_at | ZNF324 | zinc finger protein 324 | 0.0184147 | 0.396939 | 1.16515 | VAP- up vs VAP+ |
| 209365_s_at | ECM1 | extracellular matrix protein 1 | 0.0184714 | 0.396958 | 1.11032 | VAP- up vs VAP+ |
| 202598_at | S100A13 | S100 calcium binding protein A13 | 0.0186247 | 0.398445 | 1.15725 | VAP- up vs VAP+ |
| 205931_s_at | CREB5 | cAMP responsive element binding protein 5 | 0.0187323 | 0.398445 | -1.29289 | VAP- down vs VAP+ |
| 200005_at | EIF3D | eukaryotic translation initiation factor 3, subunit D | 0.0187347 | 0.398445 | 1.2129 | VAP- up vs VAP+ |
| 214596_at | CHRM3 | cholinergic receptor, muscarinic 3 | 0.0188588 | 0.398445 | 1.13589 | VAP- up vs VAP+ |
| 208677_s_at | BSG | basigin (Ok blood group) | 0.0188795 | 0.398445 | -1.38619 | VAP- down vs VAP+ |
| 217785_s_at | YKT6 | YKT6 v-SNARE homolog (S. cerevisiae) | 0.0188869 | 0.398445 | 1.14856 | VAP- up vs VAP+ |
| 202475_at | TMEM147 | transmembrane protein 147 | 0.0189865 | 0.398445 | 1.1723 | VAP- up vs VAP+ |
| 206325_at | SERPINA6 | serpin peptidase inhibitor, clade A (alpha-1 antiproteinase, antitrypsin), membe | 0.0189887 | 0.398445 | 1.14613 | VAP- up vs VAP+ |
| 205997_at | ADAM28 | ADAM metallopeptidase domain 28 | 0.0190898 | 0.399389 | -1.24074 | VAP- down vs VAP+ |
| 39548_at | NPAS2 | neuronal PAS domain protein 2 | 0.019242 | 0.400703 | -1.07964 | VAP- down vs VAP+ |
| 205663_at | PCBP3 | poly(rC) binding protein 3 | 0.0192653 | 0.400703 | 1.1135 | VAP- up vs VAP+ |
| 214299_at | TOP3A | topoisomerase (DNA) III alpha | 0.0196573 | 0.406108 | 1.11944 | VAP- up vs VAP+ |
| 200081_s_at | RPS6 | ribosomal protein S6 | 0.0196938 | 0.406108 | 1.49813 | VAP- up vs VAP+ |
| 221389_at | PLA2G2E | phospholipase A2, group IIE | 0.019709 | 0.406108 | 1.23585 | VAP- up vs VAP+ |
| 205983_at | DPEP1 | dipeptidase 1 (renal) | 0.0197535 | 0.406108 | 1.12758 | VAP- up vs VAP+ |
| 208925_at | CLDND1 | claudin domain containing 1 | 0.0199477 | 0.408007 | 1.27396 | VAP- up vs VAP+ |
| 210254_at | MS4A3 | membrane-spanning 4-domains, subfamily A, member 3 (hematopoietic cell-specific) | 0.0199606 | 0.408007 | -1.38948 | VAP- down vs VAP+ |
| 202671_s_at | PDXK | pyridoxal (pyridoxine, vitamin B6) kinase | 0.0201735 | 0.409477 | 1.41176 | VAP- up vs VAP+ |
| 204636_at | COL17A1 | collagen, type XVII, alpha 1 | 0.0201885 | 0.409477 | -1.22425 | VAP- down vs VAP+ |
| 202768_at | FOSB | FBJ murine osteosarcoma viral oncogene homolog B | 0.0202565 | 0.409477 | 1.61186 | VAP- up vs VAP+ |
| 207154_at | DIO3 | deiodinase, iodothyronine, type III | 0.0202739 | 0.409477 | 1.14946 | VAP- up vs VAP+ |
| 206978_at | CCR2 | chemokine (C-C motif) receptor 2 | 0.0203254 | 0.409477 | 1.14516 | VAP- up vs VAP+ |
| 201281_at | ADRM1 | adhesion regulating molecule 1 | 0.020404 | 0.409477 | 1.29868 | VAP- up vs VAP+ |
| 203230_at | DVL1 | dishevelled, dsh homolog 1 (Drosophila) | 0.0206856 | 0.409477 | 1.16235 | VAP- up vs VAP+ |
| 207302_at | SGCG | sarcoglycan, gamma (35kDa dystrophin-associated glycoprotein) | 0.0207329 | 0.409477 | -1.20914 | VAP- down vs VAP+ |
| 217969_at | C11orf2 | chromosome 11 open reading frame 2 | 0.0207402 | 0.409477 | 1.29872 | VAP- up vs VAP+ |
| 221425_s_at | ISCA1 | iron-sulfur cluster assembly 1 homolog (S. cerevisiae) | 0.0207824 | 0.409477 | 1.10085 | VAP- up vs VAP+ |
| 200867_at | RNF114 | ring finger protein 114 | 0.0207948 | 0.409477 | 1.10939 | VAP- up vs VAP+ |
| 202790_at | CLDN7 | claudin 7 | 0.0208016 | 0.409477 | 1.08379 | VAP- up vs VAP+ |
| 203530_s_at | STX4 | syntaxin 4 | 0.0208366 | 0.409477 | 1.48659 | VAP- up vs VAP+ |
| 220844_at | TCEB3B | transcription elongation factor B polypeptide 3B (elongin A2) | 0.0210302 | 0.409477 | -1.06218 | VAP- down vs VAP+ |
| 214457_at | HOXA2 | homeobox A2 | 0.0210672 | 0.409477 | -1.12448 | VAP- down vs VAP+ |
| 209496_at | RARRES2 | retinoic acid receptor responder (tazarotene induced) 2 | 0.0212225 | 0.409477 | 1.06644 | VAP- up vs VAP+ |
| 202908_at | WFS1 | Wolfram syndrome 1 (wolframin) | 0.0212475 | 0.409477 | 1.20853 | VAP- up vs VAP+ |
| 202891_at | NIT1 | nitrilase 1 | 0.0212791 | 0.409477 | 1.21393 | VAP- up vs VAP+ |
| 203276_at | LMNB1 | lamin B1 | 0.0213049 | 0.409477 | 1.46387 | VAP- up vs VAP+ |
| 217716_s_at | SEC61A1 | Sec61 alpha 1 subunit (S. cerevisiae) | 0.0213576 | 0.409477 | 1.28292 | VAP- up vs VAP+ |
| 204560_at | FKBP5 | FK506 binding protein 5 | 0.0214084 | 0.409477 | -1.72573 | VAP- down vs VAP+ |
| 208111_at | AVPR2 | arginine vasopressin receptor 2 | 0.0214776 | 0.409477 | 1.17829 | VAP- up vs VAP+ |
| AFFX-r2-Bs-lys-M_at | --- | --- | 0.0215847 | 0.409477 | 1.07284 | VAP- up vs VAP+ |
| 220894_x_at | PRDM12 | PR domain containing 12 | 0.0215899 | 0.409477 | 1.26286 | VAP- up vs VAP+ |
| 219244_s_at | MRPL46 | mitochondrial ribosomal protein L46 | 0.0215957 | 0.409477 | 1.10556 | VAP- up vs VAP+ |
| 217041_at | NPTXR | neuronal pentraxin receptor | 0.0216222 | 0.409477 | 1.12279 | VAP- up vs VAP+ |
| 204096_s_at | ELL | elongation factor RNA polymerase II | 0.0216749 | 0.409477 | 1.24645 | VAP- up vs VAP+ |
| 210414_at | FLRT1 | fibronectin leucine rich transmembrane protein 1 | 0.0216992 | 0.409477 | 1.11834 | VAP- up vs VAP+ |
| 200763_s_at | RPLP1 | ribosomal protein, large, P1 | 0.021795 | 0.409477 | 1.33792 | VAP- up vs VAP+ |
| 213524_s_at | G0S2 | G0/G1switch 2 | 0.0219043 | 0.409477 | 1.24292 | VAP- up vs VAP+ |
| 218497_s_at | RNASEH1 | ribonuclease H1 | 0.0219107 | 0.409477 | 1.16418 | VAP- up vs VAP+ |
| 221989_at | RPL10 | ribosomal protein L10 | 0.0219237 | 0.409477 | 1.47809 | VAP- up vs VAP+ |
| 206982_at | CRYBA1 | crystallin, beta A1 | 0.0219676 | 0.409477 | -1.18642 | VAP- down vs VAP+ |
| 205382_s_at | CFD | complement factor D (adipsin) | 0.0220343 | 0.409477 | 2.36627 | VAP- up vs VAP+ |
| 206723_s_at | LPAR2 | lysophosphatidic acid receptor 2 | 0.0220492 | 0.409477 | 1.21654 | VAP- up vs VAP+ |
| 201393_s_at | IGF2R | insulin-like growth factor 2 receptor | 0.0221373 | 0.409477 | -1.40149 | VAP- down vs VAP+ |
| 204343_at | ABCA3 | ATP-binding cassette, sub-family A (ABC1), member 3 | 0.0221624 | 0.409477 | 1.10859 | VAP- up vs VAP+ |
| 220895_at | USP29 | ubiquitin specific peptidase 29 | 0.0222685 | 0.410371 | 1.07438 | VAP- up vs VAP+ |
| 220100_at | SLC22A11 | solute carrier family 22 (organic anion/urate transporter), member 11 | 0.0223638 | 0.410922 | -1.2092 | VAP- down vs VAP+ |
| 203569_s_at | OFD1 | oral-facial-digital syndrome 1 | 0.0224139 | 0.410922 | 1.12577 | VAP- up vs VAP+ |
| 214114_x_at | FASTK | Fas-activated serine/threonine kinase | 0.0225913 | 0.413018 | 1.1335 | VAP- up vs VAP+ |
| 208383_s_at | PCK1 | phosphoenolpyruvate carboxykinase 1 (soluble) | 0.0230175 | 0.413018 | -1.11125 | VAP- down vs VAP+ |
| 320_at | PEX6 | peroxisomal biogenesis factor 6 | 0.0230185 | 0.413018 | 1.12228 | VAP- up vs VAP+ |
| 221373_x_at | PSPN | persephin | 0.0231135 | 0.413018 | 1.13925 | VAP- up vs VAP+ |
| 220525_s_at | AUP1 | ancient ubiquitous protein 1 | 0.0231142 | 0.413018 | 1.22204 | VAP- up vs VAP+ |
| 208533_at | SOX1 | SRY (sex determining region Y)-box 1 | 0.0232001 | 0.413018 | 1.09531 | VAP- up vs VAP+ |
| 202477_s_at | TUBGCP2 | tubulin, gamma complex associated protein 2 | 0.0232135 | 0.413018 | 1.23744 | VAP- up vs VAP+ |
| 203980_at | FABP4 | fatty acid binding protein 4, adipocyte | 0.0233196 | 0.413018 | 1.09403 | VAP- up vs VAP+ |
| 219735_s_at | TFCP2L1 | transcription factor CP2-like 1 | 0.0233243 | 0.413018 | 1.16773 | VAP- up vs VAP+ |
| 200937_s_at | RPL5 | ribosomal protein L5 | 0.0233894 | 0.413018 | 1.44765 | VAP- up vs VAP+ |
| 202018_s_at | LTF | lactotransferrin | 0.0233933 | 0.413018 | -2.07237 | VAP- down vs VAP+ |
| 205665_at | TSPAN9 | tetraspanin 9 | 0.0234138 | 0.413018 | 1.09768 | VAP- up vs VAP+ |
| 207143_at | CDK6 | cyclin-dependent kinase 6 | 0.0234758 | 0.413018 | -1.27155 | VAP- down vs VAP+ |
| 40446_at | PHF1 | PHD finger protein 1 | 0.0235267 | 0.413018 | 1.32458 | VAP- up vs VAP+ |
| 202376_at | SERPINA3 | serpin peptidase inhibitor, clade A (alpha-1 antiproteinase, antitrypsin), membe | 0.0235348 | 0.413018 | 1.08965 | VAP- up vs VAP+ |
| 200893_at | TRA2B | transformer 2 beta homolog (Drosophila) | 0.0236849 | 0.413018 | 1.30761 | VAP- up vs VAP+ |
| 216199_s_at | MAP3K4 | mitogen-activated protein kinase kinase kinase 4 | 0.0237796 | 0.413018 | 1.35648 | VAP- up vs VAP+ |
| 212167_s_at | SMARCB1 | SWI/SNF related, matrix associated, actin dependent regulator of chromatin, subf | 0.0238224 | 0.413018 | 1.13192 | VAP- up vs VAP+ |
| 207494_s_at | ZNF76 | zinc finger protein 76 (expressed in testis) | 0.0238308 | 0.413018 | 1.16451 | VAP- up vs VAP+ |
| 206812_at | ADRB3 | adrenergic, beta-3-, receptor | 0.0238523 | 0.413018 | -1.14697 | VAP- down vs VAP+ |
| 221351_at | HTR1A | 5-hydroxytryptamine (serotonin) receptor 1A | 0.0239909 | 0.413018 | -1.12383 | VAP- down vs VAP+ |
| 206463_s_at | DHRS2 | dehydrogenase/reductase (SDR family) member 2 | 0.0240018 | 0.413018 | 1.17402 | VAP- up vs VAP+ |
| 206970_at | CNTN2 | contactin 2 (axonal) | 0.0240366 | 0.413018 | 1.09847 | VAP- up vs VAP+ |
| 218574_s_at | LMCD1 | LIM and cysteine-rich domains 1 | 0.0241467 | 0.413018 | 1.1155 | VAP- up vs VAP+ |
| 211848_s_at | CEACAM7 | carcinoembryonic antigen-related cell adhesion molecule 7 | 0.0241629 | 0.413018 | -1.19174 | VAP- down vs VAP+ |
| 215836_s_at | PCDHGA1 /// PCDHGA10 /// PCDHGA11 /// PCDHGA12 /// PCDHGA2 /// PCDHGA3 /// PCDHGA4 /// PCDHGA5 /// PCDHGA6 /// PCDHGA7 /// PCDHGA8 /// PCDHGA9 /// PCDHGB1 /// PCDHGB2 /// PCDHGB3 /// PCDHGB4 /// PCDHGB5 /// PCDHGB6 /// PCDHGB7 /// PCDHGC3 /// PCDHGC4 /// PCDHGC5 | protocadherin gamma subfamily A, 1 /// protocadherin gamma subfamily A, 10 /// p | 0.0242477 | 0.413018 | 1.2217 | VAP- up vs VAP+ |
| 210888_s_at | ITIH1 | inter-alpha (globulin) inhibitor H1 | 0.0242527 | 0.413018 | 1.11752 | VAP- up vs VAP+ |
| 204159_at | CDKN2C | cyclin-dependent kinase inhibitor 2C (p18, inhibits CDK4) | 0.0243638 | 0.413018 | -1.13799 | VAP- down vs VAP+ |
| 203406_at | MFAP1 | microfibrillar-associated protein 1 | 0.0244117 | 0.413018 | 1.28594 | VAP- up vs VAP+ |
| 205283_at | FKTN | fukutin | 0.024444 | 0.413018 | -1.17273 | VAP- down vs VAP+ |
| 220207_at | YIF1B | Yip1 interacting factor homolog B (S. cerevisiae) | 0.0244731 | 0.413018 | -1.17873 | VAP- down vs VAP+ |
| 204352_at | TRAF5 | TNF receptor-associated factor 5 | 0.0244744 | 0.413018 | 1.18502 | VAP- up vs VAP+ |
| 221154_at | TRIM49 | tripartite motif-containing 49 | 0.024756 | 0.413018 | -1.11035 | VAP- down vs VAP+ |
| 210215_at | TFR2 | transferrin receptor 2 | 0.0247719 | 0.413018 | 1.11382 | VAP- up vs VAP+ |
| 206175_x_at | ZNF222 | zinc finger protein 222 | 0.024828 | 0.413018 | 1.26102 | VAP- up vs VAP+ |
| 221156_x_at | CCPG1 | cell cycle progression 1 | 0.0248417 | 0.413018 | -1.40253 | VAP- down vs VAP+ |
| 216083_s_at | NEU3 | sialidase 3 (membrane sialidase) | 0.0248564 | 0.413018 | 1.14657 | VAP- up vs VAP+ |
| 201252_at | PSMC4 | proteasome (prosome, macropain) 26S subunit, ATPase, 4 | 0.0249332 | 0.413018 | 1.23888 | VAP- up vs VAP+ |
| 218220_at | C12orf10 | chromosome 12 open reading frame 10 | 0.0250462 | 0.413018 | 1.1027 | VAP- up vs VAP+ |
| 210849_s_at | VPS41 | vacuolar protein sorting 41 homolog (S. cerevisiae) | 0.0250683 | 0.413018 | 1.12368 | VAP- up vs VAP+ |
| 210189_at | HSPA1L | heat shock 70kDa protein 1-like | 0.0250767 | 0.413018 | -1.21166 | VAP- down vs VAP+ |
| 218623_at | HMP19 | HMP19 protein | 0.0250773 | 0.413018 | -1.20983 | VAP- down vs VAP+ |
| 222258_s_at | SH3BP4 | SH3-domain binding protein 4 | 0.0251123 | 0.413018 | -1.13274 | VAP- down vs VAP+ |
| 205004_at | NKRF | NFKB repressing factor | 0.0251737 | 0.413018 | 1.15017 | VAP- up vs VAP+ |
| 213587_s_at | ATP6V0E2 | ATPase, H+ transporting V0 subunit e2 | 0.0252797 | 0.413018 | 1.11526 | VAP- up vs VAP+ |
| 201716_at | SNX1 | sorting nexin 1 | 0.025347 | 0.413018 | -1.161 | VAP- down vs VAP+ |
| 216836_s_at | ERBB2 | v-erb-b2 erythroblastic leukemia viral oncogene homolog 2, neuro/glioblastoma de | 0.0253485 | 0.413018 | 1.14354 | VAP- up vs VAP+ |
| 210127_at | RAB6B | RAB6B, member RAS oncogene family | 0.0253614 | 0.413018 | 1.13663 | VAP- up vs VAP+ |
| 204490_s_at | CD44 | CD44 molecule (Indian blood group) | 0.0253733 | 0.413018 | 1.24703 | VAP- up vs VAP+ |
| 200820_at | PSMD8 | proteasome (prosome, macropain) 26S subunit, non-ATPase, 8 | 0.0255686 | 0.415247 | 1.25377 | VAP- up vs VAP+ |
| 201756_at | RPA2 | replication protein A2, 32kDa | 0.0260462 | 0.419586 | 1.15434 | VAP- up vs VAP+ |
| 202138_x_at | AIMP2 | aminoacyl tRNA synthetase complex-interacting multifunctional protein 2 | 0.0260812 | 0.419586 | 1.15922 | VAP- up vs VAP+ |
| 205357_s_at | AGTR1 | angiotensin II receptor, type 1 | 0.0261811 | 0.419586 | 1.09905 | VAP- up vs VAP+ |
| 208031_s_at | RFX2 | regulatory factor X, 2 (influences HLA class II expression) | 0.0261894 | 0.419586 | -1.21648 | VAP- down vs VAP+ |
| 218401_s_at | ZNF281 | zinc finger protein 281 | 0.0262001 | 0.419586 | -1.43039 | VAP- down vs VAP+ |
| 202496_at | EDC4 | enhancer of mRNA decapping 4 | 0.0264081 | 0.419586 | 1.16208 | VAP- up vs VAP+ |
| 211339_s_at | ITK | IL2-inducible T-cell kinase | 0.0264835 | 0.419586 | 1.49995 | VAP- up vs VAP+ |
| 203986_at | STBD1 | starch binding domain 1 | 0.0264844 | 0.419586 | -1.17432 | VAP- down vs VAP+ |
| 210523_at | BMPR1B | bone morphogenetic protein receptor, type IB | 0.0265042 | 0.419586 | -1.25403 | VAP- down vs VAP+ |
| 221112_at | IL1RAPL2 | interleukin 1 receptor accessory protein-like 2 | 0.0265332 | 0.419586 | -1.1516 | VAP- down vs VAP+ |
| 203167_at | TIMP2 | TIMP metallopeptidase inhibitor 2 | 0.0265386 | 0.419586 | -1.2374 | VAP- down vs VAP+ |
| 1487_at | ESRRA | estrogen-related receptor alpha | 0.0266397 | 0.419586 | 1.16604 | VAP- up vs VAP+ |
| 200022_at | RPL18 | ribosomal protein L18 | 0.0266909 | 0.419586 | 1.43694 | VAP- up vs VAP+ |
| 200910_at | CCT3 | chaperonin containing TCP1, subunit 3 (gamma) | 0.0267144 | 0.419586 | 1.37254 | VAP- up vs VAP+ |
| 221260_s_at | CSRNP2 | cysteine-serine-rich nuclear protein 2 | 0.0267206 | 0.419586 | -1.20939 | VAP- down vs VAP+ |
| 201596_x_at | KRT18 | keratin 18 | 0.0269743 | 0.421321 | 1.18523 | VAP- up vs VAP+ |
| 206801_at | NPPB | natriuretic peptide precursor B | 0.0270011 | 0.421321 | 1.09901 | VAP- up vs VAP+ |
| 214464_at | CDC42BPA | CDC42 binding protein kinase alpha (DMPK-like) | 0.0270369 | 0.421321 | 1.11793 | VAP- up vs VAP+ |
| 50277_at | GGA1 | golgi-associated, gamma adaptin ear containing, ARF binding protein 1 | 0.0271941 | 0.421321 | 1.20691 | VAP- up vs VAP+ |
| 219638_at | FBXO22 | F-box protein 22 | 0.0272 | 0.421321 | -1.17293 | VAP- down vs VAP+ |
| 205428_s_at | CALB2 | calbindin 2 | 0.0272378 | 0.421321 | 1.11689 | VAP- up vs VAP+ |
| 202121_s_at | CHMP2A | chromatin modifying protein 2A | 0.0273112 | 0.421321 | 1.3183 | VAP- up vs VAP+ |
| 209003_at | SLC25A11 | solute carrier family 25 (mitochondrial carrier; oxoglutarate carrier), member 1 | 0.0273491 | 0.421321 | 1.30309 | VAP- up vs VAP+ |
| 65588_at | LOC388796 | hypothetical LOC388796 | 0.0273945 | 0.421321 | 1.12435 | VAP- up vs VAP+ |
| 207673_at | NPHS1 | nephrosis 1, congenital, Finnish type (nephrin) | 0.0274576 | 0.421321 | -1.09802 | VAP- down vs VAP+ |
| 210160_at | PAFAH1B2 | platelet-activating factor acetylhydrolase 1b, catalytic subunit 2 (30kDa) | 0.0274826 | 0.421321 | -1.12542 | VAP- down vs VAP+ |
| 220519_s_at | LIM2 | lens intrinsic membrane protein 2, 19kDa | 0.0275625 | 0.421331 | -1.15898 | VAP- down vs VAP+ |
| 204959_at | MNDA | myeloid cell nuclear differentiation antigen | 0.0276017 | 0.421331 | -1.94488 | VAP- down vs VAP+ |
| 206954_at | WIT1 | Wilms tumor upstream neighbor 1 | 0.0277063 | 0.422022 | -1.12319 | VAP- down vs VAP+ |
| 202191_s_at | GAS7 | growth arrest-specific 7 | 0.027807 | 0.422619 | -1.72055 | VAP- down vs VAP+ |
| 209185_s_at | IRS2 | insulin receptor substrate 2 | 0.0279643 | 0.422619 | -1.63913 | VAP- down vs VAP+ |
| 1729_at | TRADD | TNFRSF1A-associated via death domain | 0.0280418 | 0.422619 | 1.14407 | VAP- up vs VAP+ |
| 205226_at | PDGFRL | platelet-derived growth factor receptor-like | 0.0280477 | 0.422619 | 1.11355 | VAP- up vs VAP+ |
| 220188_at | JPH3 | junctophilin 3 | 0.0281254 | 0.422619 | 1.0708 | VAP- up vs VAP+ |
| 207541_s_at | EXOSC10 | exosome component 10 | 0.0281572 | 0.422619 | 1.16061 | VAP- up vs VAP+ |
| 40420_at | STK10 | serine/threonine kinase 10 | 0.0281829 | 0.422619 | 1.31582 | VAP- up vs VAP+ |
| 201011_at | RPN1 | ribophorin I | 0.0282222 | 0.422619 | 1.41461 | VAP- up vs VAP+ |
| 209635_at | AP1S1 | adaptor-related protein complex 1, sigma 1 subunit | 0.0282802 | 0.422619 | -1.19976 | VAP- down vs VAP+ |
| 214375_at | LOC729222 /// PPFIBP1 | similar to PTPRF interacting protein binding protein 1 /// PTPRF interacting pro | 0.0283966 | 0.422647 | 1.1128 | VAP- up vs VAP+ |
| 221160_s_at | CABP5 | calcium binding protein 5 | 0.0284522 | 0.422647 | 1.12812 | VAP- up vs VAP+ |
| 206002_at | GPR64 | G protein-coupled receptor 64 | 0.0286303 | 0.422647 | -1.13829 | VAP- down vs VAP+ |
| 203411_s_at | LMNA | lamin A/C | 0.0287254 | 0.422647 | 1.25559 | VAP- up vs VAP+ |
| 222030_at | SIVA1 | SIVA1, apoptosis-inducing factor | 0.0287605 | 0.422647 | 1.21231 | VAP- up vs VAP+ |
| 201379_s_at | TPD52L2 | tumor protein D52-like 2 | 0.0287797 | 0.422647 | 1.33343 | VAP- up vs VAP+ |
| 202159_at | FARSA | phenylalanyl-tRNA synthetase, alpha subunit | 0.0288287 | 0.422647 | 1.20054 | VAP- up vs VAP+ |
| 205726_at | DIAPH2 | diaphanous homolog 2 (Drosophila) | 0.028838 | 0.422647 | -1.17189 | VAP- down vs VAP+ |
| 202806_at | DBN1 | drebrin 1 | 0.0289084 | 0.422647 | 1.18972 | VAP- up vs VAP+ |
| 204602_at | DKK1 | dickkopf homolog 1 (Xenopus laevis) | 0.0289538 | 0.422647 | -1.11271 | VAP- down vs VAP+ |
| AFFX-M27830_5_at | --- | --- | 0.0290228 | 0.422647 | 1.18929 | VAP- up vs VAP+ |
| 218157_x_at | CDC42SE1 | CDC42 small effector 1 | 0.0290509 | 0.422647 | 1.19642 | VAP- up vs VAP+ |
| 207628_s_at | WBSCR22 | Williams Beuren syndrome chromosome region 22 | 0.0290545 | 0.422647 | 1.18799 | VAP- up vs VAP+ |
| 204478_s_at | RABIF | RAB interacting factor | 0.0291736 | 0.423513 | -1.19471 | VAP- down vs VAP+ |
| 206367_at | REN | renin | 0.0293753 | 0.424173 | 1.12741 | VAP- up vs VAP+ |
| 38043_at | FAM3A | family with sequence similarity 3, member A | 0.0294357 | 0.424173 | 1.10708 | VAP- up vs VAP+ |
| 220408_x_at | FAM48A | family with sequence similarity 48, member A | 0.0294488 | 0.424173 | 1.18399 | VAP- up vs VAP+ |
| 203484_at | SEC61G | Sec61 gamma subunit | 0.0295275 | 0.424173 | 1.49175 | VAP- up vs VAP+ |
| 219305_x_at | FBXO2 | F-box protein 2 | 0.029601 | 0.424173 | 1.0993 | VAP- up vs VAP+ |
| 205695_at | SDS | serine dehydratase | 0.0297531 | 0.424173 | 1.12794 | VAP- up vs VAP+ |
| 207363_at | RS1 | retinoschisin 1 | 0.0298117 | 0.424173 | -1.12729 | VAP- down vs VAP+ |
| 204144_s_at | PIGQ | phosphatidylinositol glycan anchor biosynthesis, class Q | 0.029812 | 0.424173 | 1.1278 | VAP- up vs VAP+ |
| 209102_s_at | HBP1 | HMG-box transcription factor 1 | 0.0298202 | 0.424173 | -1.30689 | VAP- down vs VAP+ |
| 218457_s_at | DNMT3A | DNA (cytosine-5-)-methyltransferase 3 alpha | 0.0299885 | 0.424173 | 1.15449 | VAP- up vs VAP+ |
| 204082_at | PBX3 | pre-B-cell leukemia homeobox 3 | 0.0299957 | 0.424173 | -1.13122 | VAP- down vs VAP+ |
| 200965_s_at | ABLIM1 | actin binding LIM protein 1 | 0.0301061 | 0.424173 | 1.33416 | VAP- up vs VAP+ |
| 208572_at | HIST3H3 | histone cluster 3, H3 | 0.0301091 | 0.424173 | 1.11639 | VAP- up vs VAP+ |
| 221383_at | NMUR1 | neuromedin U receptor 1 | 0.0302282 | 0.424173 | 1.12063 | VAP- up vs VAP+ |
| 209369_at | ANXA3 | annexin A3 | 0.0302672 | 0.424173 | -1.80127 | VAP- down vs VAP+ |
| 205633_s_at | ALAS1 | aminolevulinate, delta-, synthase 1 | 0.0303333 | 0.424173 | -1.36156 | VAP- down vs VAP+ |
| 220510_at | RHBG | Rh family, B glycoprotein (gene/pseudogene) | 0.0304429 | 0.424173 | 1.20445 | VAP- up vs VAP+ |
| 214549_x_at | SPRR1A | small proline-rich protein 1A | 0.0304626 | 0.424173 | 1.16543 | VAP- up vs VAP+ |
| 205255_x_at | TCF7 | transcription factor 7 (T-cell specific, HMG-box) | 0.0305068 | 0.424173 | 1.35614 | VAP- up vs VAP+ |
| 221114_at | AMBN | ameloblastin (enamel matrix protein) | 0.0305966 | 0.424173 | -1.16082 | VAP- down vs VAP+ |
| 219553_at | NME7 | non-metastatic cells 7, protein expressed in (nucleoside-diphosphate kinase) | 0.0306492 | 0.424173 | 1.11361 | VAP- up vs VAP+ |
| 205946_at | VIPR2 | vasoactive intestinal peptide receptor 2 | 0.0306958 | 0.424173 | 1.12786 | VAP- up vs VAP+ |
| 219287_at | KCNMB4 | potassium large conductance calcium-activated channel, subfamily M, beta member | 0.0307155 | 0.424173 | -1.12347 | VAP- down vs VAP+ |
| 210311_at | FGF5 | fibroblast growth factor 5 | 0.0307652 | 0.424173 | 1.14224 | VAP- up vs VAP+ |
| 208904_s_at | RPS28 | ribosomal protein S28 | 0.0308259 | 0.424173 | 1.39991 | VAP- up vs VAP+ |
| 221266_s_at | TM7SF4 | transmembrane 7 superfamily member 4 | 0.0308275 | 0.424173 | -1.13157 | VAP- down vs VAP+ |
| 209082_s_at | COL18A1 | collagen, type XVIII, alpha 1 | 0.0308291 | 0.424173 | 1.18925 | VAP- up vs VAP+ |
| 207663_x_at | GAGE3 | G antigen 3 | 0.0308969 | 0.424286 | -1.11804 | VAP- down vs VAP+ |
| 209574_s_at | C18orf1 | chromosome 18 open reading frame 1 | 0.0311661 | 0.426111 | 1.14628 | VAP- up vs VAP+ |
| 219110_at | GAR1 | GAR1 ribonucleoprotein homolog (yeast) | 0.0311724 | 0.426111 | 1.16276 | VAP- up vs VAP+ |
| 205456_at | CD3E | CD3e molecule, epsilon (CD3-TCR complex) | 0.0312095 | 0.426111 | 1.17123 | VAP- up vs VAP+ |
| 210046_s_at | IDH2 | isocitrate dehydrogenase 2 (NADP+), mitochondrial | 0.0313579 | 0.426727 | 1.16276 | VAP- up vs VAP+ |
| 203818_s_at | SF3A3 | splicing factor 3a, subunit 3, 60kDa | 0.0313746 | 0.426727 | 1.25918 | VAP- up vs VAP+ |
| 221669_s_at | ACAD8 | acyl-CoA dehydrogenase family, member 8 | 0.0316169 | 0.429201 | -1.25427 | VAP- down vs VAP+ |
| 208207_at | --- | --- | 0.0320316 | 0.429766 | -1.10087 | VAP- down vs VAP+ |
| 207907_at | TNFSF14 | tumor necrosis factor (ligand) superfamily, member 14 | 0.032048 | 0.429766 | 1.59141 | VAP- up vs VAP+ |
| 200036_s_at | RPL10A | ribosomal protein L10a | 0.0322232 | 0.429766 | 1.46069 | VAP- up vs VAP+ |
| 206234_s_at | MMP17 | matrix metallopeptidase 17 (membrane-inserted) | 0.0322568 | 0.429766 | 1.20085 | VAP- up vs VAP+ |
| 203522_at | CCS | copper chaperone for superoxide dismutase | 0.0322885 | 0.429766 | 1.20308 | VAP- up vs VAP+ |
| 209980_s_at | SHMT1 | serine hydroxymethyltransferase 1 (soluble) | 0.0324795 | 0.429766 | 1.12894 | VAP- up vs VAP+ |
| 201272_at | AKR1B1 | aldo-keto reductase family 1, member B1 (aldose reductase) | 0.0325365 | 0.429766 | 1.15918 | VAP- up vs VAP+ |
| 220139_at | DNMT3L | DNA (cytosine-5-)-methyltransferase 3-like | 0.0326251 | 0.429766 | 1.08567 | VAP- up vs VAP+ |
| 208437_at | CLCN1 | chloride channel 1, skeletal muscle | 0.0326344 | 0.429766 | -1.19713 | VAP- down vs VAP+ |
| 204401_at | KCNN4 | potassium intermediate/small conductance calcium-activated channel, subfamily N, | 0.0326962 | 0.429766 | 1.26956 | VAP- up vs VAP+ |
| 217960_s_at | TOMM22 | translocase of outer mitochondrial membrane 22 homolog (yeast) | 0.0327356 | 0.429766 | 1.16871 | VAP- up vs VAP+ |
| 203292_s_at | VPS11 | vacuolar protein sorting 11 homolog (S. cerevisiae) | 0.0328155 | 0.429766 | 1.13344 | VAP- up vs VAP+ |
| 204260_at | CHGB | chromogranin B (secretogranin 1) | 0.0329283 | 0.429766 | 1.11044 | VAP- up vs VAP+ |
| 219285_s_at | NIN | ninein (GSK3B interacting protein) | 0.033033 | 0.429766 | -1.1331 | VAP- down vs VAP+ |
| 203564_at | FANCG | Fanconi anemia, complementation group G | 0.0331678 | 0.429766 | 1.05665 | VAP- up vs VAP+ |
| 221312_at | GLP2R | glucagon-like peptide 2 receptor | 0.0331846 | 0.429766 | -1.1503 | VAP- down vs VAP+ |
| 212318_at | TNPO3 | transportin 3 | 0.0331973 | 0.429766 | -1.23215 | VAP- down vs VAP+ |
| 208574_at | SOX14 | SRY (sex determining region Y)-box 14 | 0.0332516 | 0.429766 | -1.07132 | VAP- down vs VAP+ |
| 207864_at | SCN7A | sodium channel, voltage-gated, type VII, alpha | 0.0332538 | 0.429766 | -1.05563 | VAP- down vs VAP+ |
| 210080_x_at | CELA3A | chymotrypsin-like elastase family, member 3A | 0.0332542 | 0.429766 | 1.12674 | VAP- up vs VAP+ |
| 210143_at | ANXA10 | annexin A10 | 0.0332854 | 0.429766 | -1.23316 | VAP- down vs VAP+ |
| 214539_at | SERPINB10 | serpin peptidase inhibitor, clade B (ovalbumin), member 10 | 0.033293 | 0.429766 | -1.24002 | VAP- down vs VAP+ |
| 217807_s_at | GLTSCR2 | glioma tumor suppressor candidate region gene 2 | 0.033317 | 0.429766 | 1.53099 | VAP- up vs VAP+ |
| 200074_s_at | RPL14 | ribosomal protein L14 | 0.033325 | 0.429766 | 1.50964 | VAP- up vs VAP+ |
| 203103_s_at | PRPF19 | PRP19/PSO4 pre-mRNA processing factor 19 homolog (S. cerevisiae) | 0.0333517 | 0.429766 | 1.14582 | VAP- up vs VAP+ |
| 207716_at | KRT38 | keratin 38 | 0.0334069 | 0.429766 | -1.22901 | VAP- down vs VAP+ |
| 204554_at | PPP1R3D | protein phosphatase 1, regulatory (inhibitor) subunit 3D | 0.0334486 | 0.429766 | -1.43994 | VAP- down vs VAP+ |
| 205530_at | ETFDH | electron-transferring-flavoprotein dehydrogenase | 0.0334677 | 0.429766 | 1.11883 | VAP- up vs VAP+ |
| 207585_s_at | RPL36AL | ribosomal protein L36a-like | 0.0335291 | 0.429766 | 1.41885 | VAP- up vs VAP+ |
| 200652_at | SSR2 | signal sequence receptor, beta (translocon-associated protein beta) | 0.0335634 | 0.429766 | 1.29376 | VAP- up vs VAP+ |
| 205610_at | MYOM1 | myomesin 1, 185kDa | 0.0335815 | 0.429766 | -1.15471 | VAP- down vs VAP+ |
| 201956_s_at | GNPAT | glyceronephosphate O-acyltransferase | 0.0335918 | 0.429766 | -1.22365 | VAP- down vs VAP+ |
| 208250_s_at | DMBT1 | deleted in malignant brain tumors 1 | 0.0337598 | 0.43114 | 1.13436 | VAP- up vs VAP+ |
| 205486_at | TESK2 | testis-specific kinase 2 | 0.0339585 | 0.431359 | 1.22716 | VAP- up vs VAP+ |
| 212401_s_at | CDK11A | cyclin-dependent kinase 11A | 0.0339619 | 0.431359 | 1.2729 | VAP- up vs VAP+ |
| 220970_s_at | KRTAP2-4 | keratin associated protein 2-4 | 0.0340353 | 0.431359 | 1.09781 | VAP- up vs VAP+ |
| 207372_s_at | ENTPD2 | ectonucleoside triphosphate diphosphohydrolase 2 | 0.0341479 | 0.431359 | -1.07549 | VAP- down vs VAP+ |
| 211285_s_at | UBE3A | ubiquitin protein ligase E3A | 0.0341569 | 0.431359 | 1.29143 | VAP- up vs VAP+ |
| 215498_s_at | MAP2K3 | mitogen-activated protein kinase kinase 3 | 0.0342889 | 0.431359 | 1.21747 | VAP- up vs VAP+ |
| 204849_at | TCFL5 | transcription factor-like 5 (basic helix-loop-helix) | 0.0343328 | 0.431359 | 1.80482 | VAP- up vs VAP+ |
| 200807_s_at | HSPD1 | heat shock 60kDa protein 1 (chaperonin) | 0.0345041 | 0.431359 | 1.39894 | VAP- up vs VAP+ |
| 207936_x_at | RFPL3 | ret finger protein-like 3 | 0.0346779 | 0.431359 | 1.09073 | VAP- up vs VAP+ |
| 220049_s_at | PDCD1LG2 | programmed cell death 1 ligand 2 | 0.0347267 | 0.431359 | 1.16349 | VAP- up vs VAP+ |
| 203579_s_at | SLC7A6 | solute carrier family 7 (cationic amino acid transporter, y+ system), member 6 | 0.0350493 | 0.431359 | 1.06776 | VAP- up vs VAP+ |
| 40284_at | FOXA2 | forkhead box A2 | 0.0351899 | 0.431359 | 1.07644 | VAP- up vs VAP+ |
| 200823_x_at | RPL29 | ribosomal protein L29 | 0.0352274 | 0.431359 | 1.4178 | VAP- up vs VAP+ |
| 217772_s_at | MTCH2 | mitochondrial carrier homolog 2 (C. elegans) | 0.0352845 | 0.431359 | 1.202 | VAP- up vs VAP+ |
| 200812_at | CCT7 | chaperonin containing TCP1, subunit 7 (eta) | 0.0353422 | 0.431359 | 1.20137 | VAP- up vs VAP+ |
| 206799_at | SCGB1D2 | secretoglobin, family 1D, member 2 | 0.0354372 | 0.431359 | -1.20779 | VAP- down vs VAP+ |
| 220571_at | PRDM11 | PR domain containing 11 | 0.0355555 | 0.431359 | 1.14083 | VAP- up vs VAP+ |
| 220821_at | GALR1 | galanin receptor 1 | 0.0356353 | 0.431359 | 1.10269 | VAP- up vs VAP+ |
| 205981_s_at | ING2 | inhibitor of growth family, member 2 | 0.0356835 | 0.431359 | -1.20429 | VAP- down vs VAP+ |
| 200834_s_at | LOC100291837 /// RPS21 | similar to ribosomal protein S21 /// ribosomal protein S21 | 0.035731 | 0.431359 | 1.38233 | VAP- up vs VAP+ |
| 220171_x_at | KIAA1704 | KIAA1704 | 0.035732 | 0.431359 | -1.07521 | VAP- down vs VAP+ |
| 219664_s_at | DECR2 | 2,4-dienoyl CoA reductase 2, peroxisomal | 0.0357477 | 0.431359 | 1.08108 | VAP- up vs VAP+ |
| 217006_x_at | FASN | fatty acid synthase | 0.0357906 | 0.431359 | 1.12719 | VAP- up vs VAP+ |
| 208009_s_at | ARHGEF16 | Rho guanine nucleotide exchange factor (GEF) 16 | 0.0357953 | 0.431359 | 1.13346 | VAP- up vs VAP+ |
| 206214_at | PLA2G7 | phospholipase A2, group VII (platelet-activating factor acetylhydrolase, plasma) | 0.0358347 | 0.431359 | 1.27436 | VAP- up vs VAP+ |
| 206613_s_at | TAF1A | TATA box binding protein (TBP)-associated factor, RNA polymerase I, A, 48kDa | 0.0358353 | 0.431359 | 1.05779 | VAP- up vs VAP+ |
| 204924_at | TLR2 | toll-like receptor 2 | 0.0358454 | 0.431359 | -1.23871 | VAP- down vs VAP+ |
| 202831_at | GPX2 | glutathione peroxidase 2 (gastrointestinal) | 0.0359395 | 0.431359 | -1.11287 | VAP- down vs VAP+ |
| 208322_s_at | ST3GAL1 | ST3 beta-galactoside alpha-2,3-sialyltransferase 1 | 0.0359752 | 0.431359 | 1.24474 | VAP- up vs VAP+ |
| 203673_at | TG | thyroglobulin | 0.0359935 | 0.431359 | -1.1983 | VAP- down vs VAP+ |
| 200031_s_at | RPS11 | ribosomal protein S11 | 0.0360636 | 0.431359 | 1.287 | VAP- up vs VAP+ |
| 200013_at | RPL24 | ribosomal protein L24 | 0.036067 | 0.431359 | 1.34357 | VAP- up vs VAP+ |
| 221049_s_at | POLL | polymerase (DNA directed), lambda | 0.0361063 | 0.431359 | 1.15132 | VAP- up vs VAP+ |
| 219579_at | RAB3IL1 | RAB3A interacting protein (rabin3)-like 1 | 0.0361103 | 0.431359 | -1.22626 | VAP- down vs VAP+ |
| 205304_s_at | KCNJ8 | potassium inwardly-rectifying channel, subfamily J, member 8 | 0.0361303 | 0.431359 | -1.26975 | VAP- down vs VAP+ |
| 200779_at | ATF4 | activating transcription factor 4 (tax-responsive enhancer element B67) | 0.0361535 | 0.431359 | 1.21713 | VAP- up vs VAP+ |
| 215116_s_at | DNM1 | dynamin 1 | 0.0361757 | 0.431359 | 1.08362 | VAP- up vs VAP+ |
| 214478_at | SPP2 | secreted phosphoprotein 2, 24kDa | 0.0361861 | 0.431359 | 1.08456 | VAP- up vs VAP+ |
| 206676_at | CEACAM8 | carcinoembryonic antigen-related cell adhesion molecule 8 | 0.0362267 | 0.431359 | -1.6193 | VAP- down vs VAP+ |
| 208311_at | GPR50 | G protein-coupled receptor 50 | 0.0362871 | 0.431359 | 1.09129 | VAP- up vs VAP+ |
| 219624_at | BAG4 | BCL2-associated athanogene 4 | 0.0362909 | 0.431359 | -1.1395 | VAP- down vs VAP+ |
| 206705_at | TULP1 | tubby like protein 1 | 0.0363239 | 0.431359 | 1.11926 | VAP- up vs VAP+ |
| 220294_at | KCNV1 | potassium channel, subfamily V, member 1 | 0.0364008 | 0.431552 | -1.12036 | VAP- down vs VAP+ |
| 200959_at | FUS | fused in sarcoma | 0.0366313 | 0.431824 | 1.1787 | VAP- up vs VAP+ |
| 214447_at | ETS1 | v-ets erythroblastosis virus E26 oncogene homolog 1 (avian) | 0.0366766 | 0.431824 | -1.19247 | VAP- down vs VAP+ |
| 219993_at | SOX17 | SRY (sex determining region Y)-box 17 | 0.0367164 | 0.431824 | -1.11168 | VAP- down vs VAP+ |
| 202012_s_at | EXT2 | exostosin 2 | 0.0369159 | 0.431824 | 1.1691 | VAP- up vs VAP+ |
| 205678_at | AP3B2 | adaptor-related protein complex 3, beta 2 subunit | 0.0369179 | 0.431824 | -1.16887 | VAP- down vs VAP+ |
| 207260_at | FEV | FEV (ETS oncogene family) | 0.0370005 | 0.431824 | -1.21957 | VAP- down vs VAP+ |
| 218629_at | SMO | smoothened homolog (Drosophila) | 0.0371448 | 0.431824 | 1.1056 | VAP- up vs VAP+ |
| 220443_s_at | VAX2 | ventral anterior homeobox 2 | 0.0372246 | 0.431824 | 1.0914 | VAP- up vs VAP+ |
| 201753_s_at | ADD3 | adducin 3 (gamma) | 0.0373198 | 0.431824 | -1.49339 | VAP- down vs VAP+ |
| 202286_s_at | TACSTD2 | tumor-associated calcium signal transducer 2 | 0.0373449 | 0.431824 | -1.20927 | VAP- down vs VAP+ |
| 201530_x_at | EIF4A1 | eukaryotic translation initiation factor 4A1 | 0.037424 | 0.431824 | 1.243 | VAP- up vs VAP+ |
| 203221_at | TLE1 | transducin-like enhancer of split 1 (E(sp1) homolog, Drosophila) | 0.0374899 | 0.431824 | -1.29244 | VAP- down vs VAP+ |
| 209199_s_at | MEF2C | myocyte enhancer factor 2C | 0.0375827 | 0.431824 | 1.18407 | VAP- up vs VAP+ |
| 206565_x_at | SMA4 | glucuronidase, beta pseudogene | 0.0376148 | 0.431824 | -1.47541 | VAP- down vs VAP+ |
| 218078_s_at | ZDHHC3 | zinc finger, DHHC-type containing 3 | 0.0376439 | 0.431824 | -1.38982 | VAP- down vs VAP+ |
| 205978_at | KL | klotho | 0.0377413 | 0.431824 | -1.15933 | VAP- down vs VAP+ |
| 202912_at | ADM | adrenomedullin | 0.0377608 | 0.431824 | 1.23447 | VAP- up vs VAP+ |
| 218432_at | FBXO3 | F-box protein 3 | 0.0377747 | 0.431824 | -1.12465 | VAP- down vs VAP+ |
| 211145_x_at | IFNA21 | interferon, alpha 21 | 0.0379414 | 0.431824 | -1.16441 | VAP- down vs VAP+ |
| 219666_at | MS4A6A | membrane-spanning 4-domains, subfamily A, member 6A | 0.0379606 | 0.431824 | -1.24316 | VAP- down vs VAP+ |
| 205024_s_at | RAD51 | RAD51 homolog (RecA homolog, E. coli) (S. cerevisiae) | 0.0380177 | 0.431824 | -1.13582 | VAP- down vs VAP+ |
| 214490_at | ARSF | arylsulfatase F | 0.0381205 | 0.431824 | 1.08609 | VAP- up vs VAP+ |
| 218634_at | PHLDA3 | pleckstrin homology-like domain, family A, member 3 | 0.0382945 | 0.431824 | 1.14161 | VAP- up vs VAP+ |
| 202690_s_at | SNRPD1 | small nuclear ribonucleoprotein D1 polypeptide 16kDa | 0.0383112 | 0.431824 | 1.21799 | VAP- up vs VAP+ |
| 203916_at | NDST2 | N-deacetylase/N-sulfotransferase (heparan glucosaminyl) 2 | 0.0384124 | 0.431824 | -1.1382 | VAP- down vs VAP+ |
| 207064_s_at | AOC2 | amine oxidase, copper containing 2 (retina-specific) | 0.0384922 | 0.431824 | 1.1075 | VAP- up vs VAP+ |
| 205052_at | AUH | AU RNA binding protein/enoyl-CoA hydratase | 0.0385829 | 0.431824 | 1.07689 | VAP- up vs VAP+ |
| 208650_s_at | CD24 | CD24 molecule | 0.0386186 | 0.431824 | -1.53191 | VAP- down vs VAP+ |
| 220847_x_at | ZNF221 | zinc finger protein 221 | 0.0386653 | 0.431824 | 1.09695 | VAP- up vs VAP+ |
| 212266_s_at | SFRS5 | splicing factor, arginine/serine-rich 5 | 0.0387047 | 0.431824 | 1.20646 | VAP- up vs VAP+ |
| 220807_at | HBQ1 | hemoglobin, theta 1 | 0.0387162 | 0.431824 | 1.27367 | VAP- up vs VAP+ |
| 210993_s_at | SMAD1 | SMAD family member 1 | 0.0387164 | 0.431824 | -1.22615 | VAP- down vs VAP+ |
| 206754_s_at | CYP2B6 /// CYP2B7P1 | cytochrome P450, family 2, subfamily B, polypeptide 6 /// cytochrome P450, famil | 0.038813 | 0.431824 | 1.24763 | VAP- up vs VAP+ |
| 210058_at | MAPK13 | mitogen-activated protein kinase 13 | 0.0388506 | 0.431824 | 1.26755 | VAP- up vs VAP+ |
| 220005_at | P2RY13 | purinergic receptor P2Y, G-protein coupled, 13 | 0.0388749 | 0.431824 | -1.36895 | VAP- down vs VAP+ |
| 205576_at | SERPIND1 | serpin peptidase inhibitor, clade D (heparin cofactor), member 1 | 0.0389678 | 0.431824 | -1.09261 | VAP- down vs VAP+ |
| 219509_at | MYOZ1 | myozenin 1 | 0.0391253 | 0.431824 | -1.16267 | VAP- down vs VAP+ |
| 208967_s_at | AK2 | adenylate kinase 2 | 0.0391677 | 0.431824 | 1.24015 | VAP- up vs VAP+ |
| 209336_at | PWP2 | PWP2 periodic tryptophan protein homolog (yeast) | 0.039208 | 0.431824 | 1.08143 | VAP- up vs VAP+ |
| 201149_s_at | TIMP3 | TIMP metallopeptidase inhibitor 3 | 0.0392122 | 0.431824 | 1.15887 | VAP- up vs VAP+ |
| 201491_at | AHSA1 | AHA1, activator of heat shock 90kDa protein ATPase homolog 1 (yeast) | 0.0394582 | 0.431824 | 1.14374 | VAP- up vs VAP+ |
| 204586_at | BSN | bassoon (presynaptic cytomatrix protein) | 0.0394917 | 0.431824 | -1.21313 | VAP- down vs VAP+ |
| 208658_at | PDIA4 | protein disulfide isomerase family A, member 4 | 0.0397236 | 0.431824 | 1.14783 | VAP- up vs VAP+ |
| 213755_s_at | --- | --- | 0.0397367 | 0.431824 | 1.1016 | VAP- up vs VAP+ |
| 212803_at | NAB2 | NGFI-A binding protein 2 (EGR1 binding protein 2) | 0.0397453 | 0.431824 | 1.16746 | VAP- up vs VAP+ |
| 202357_s_at | CFB | complement factor B | 0.0397826 | 0.431824 | 1.41602 | VAP- up vs VAP+ |
| 203645_s_at | CD163 | CD163 molecule | 0.0397885 | 0.431824 | -1.6085 | VAP- down vs VAP+ |
| 209123_at | QDPR | quinoid dihydropteridine reductase | 0.0399715 | 0.431824 | 1.14257 | VAP- up vs VAP+ |
| 202107_s_at | MCM2 | minichromosome maintenance complex component 2 | 0.0402839 | 0.431824 | 1.14256 | VAP- up vs VAP+ |
| 208445_s_at | BAZ1B | bromodomain adjacent to zinc finger domain, 1B | 0.0403025 | 0.431824 | 1.07885 | VAP- up vs VAP+ |
| 202348_s_at | TOR1A | torsin family 1, member A (torsin A) | 0.0403035 | 0.431824 | 1.32472 | VAP- up vs VAP+ |
| 209660_at | TTR | transthyretin | 0.0403388 | 0.431824 | 1.19341 | VAP- up vs VAP+ |
| 202842_s_at | DNAJB9 | DnaJ (Hsp40) homolog, subfamily B, member 9 | 0.0403919 | 0.431824 | 1.51774 | VAP- up vs VAP+ |
| 202506_at | SSFA2 | sperm specific antigen 2 | 0.0404592 | 0.431824 | -1.21445 | VAP- down vs VAP+ |
| 206210_s_at | CETP | cholesteryl ester transfer protein, plasma | 0.0404671 | 0.431824 | -1.22483 | VAP- down vs VAP+ |
| 206064_s_at | PPIL2 | peptidylprolyl isomerase (cyclophilin)-like 2 | 0.0405734 | 0.431824 | -1.14951 | VAP- down vs VAP+ |
| 204359_at | FLRT2 | fibronectin leucine rich transmembrane protein 2 | 0.0405773 | 0.431824 | 1.08114 | VAP- up vs VAP+ |
| 56197_at | PLSCR3 | phospholipid scramblase 3 | 0.0406215 | 0.431824 | 1.10994 | VAP- up vs VAP+ |
| 205742_at | TNNI3 | troponin I type 3 (cardiac) | 0.0407216 | 0.431824 | 1.09572 | VAP- up vs VAP+ |
| 203810_at | DNAJB4 | DnaJ (Hsp40) homolog, subfamily B, member 4 | 0.0407233 | 0.431824 | -1.15794 | VAP- down vs VAP+ |
| 200088_x_at | RPL12 | ribosomal protein L12 | 0.04087 | 0.431824 | 1.35948 | VAP- up vs VAP+ |
| 202241_at | TRIB1 | tribbles homolog 1 (Drosophila) | 0.0410116 | 0.431824 | 1.32324 | VAP- up vs VAP+ |
| 200918_s_at | SRPR | signal recognition particle receptor (docking protein) | 0.0410752 | 0.431824 | 1.19767 | VAP- up vs VAP+ |
| 206077_at | KEL | Kell blood group, metallo-endopeptidase | 0.0410901 | 0.431824 | -1.15807 | VAP- down vs VAP+ |
| 205647_at | RAD52 | RAD52 homolog (S. cerevisiae) | 0.0411184 | 0.431824 | 1.14989 | VAP- up vs VAP+ |
| 206957_at | AGXT | alanine-glyoxylate aminotransferase | 0.0411356 | 0.431824 | -1.13489 | VAP- down vs VAP+ |
| 206131_at | CLPS | colipase, pancreatic | 0.0412999 | 0.431824 | 1.10072 | VAP- up vs VAP+ |
| 206467_x_at | RTEL1 /// TNFRSF6B | regulator of telomere elongation helicase 1 /// tumor necrosis factor receptor s | 0.0413033 | 0.431824 | 1.15344 | VAP- up vs VAP+ |
| 208373_s_at | P2RY6 | pyrimidinergic receptor P2Y, G-protein coupled, 6 | 0.041446 | 0.431824 | 1.14229 | VAP- up vs VAP+ |
| 205795_at | NRXN3 | neurexin 3 | 0.0414877 | 0.431824 | -1.10843 | VAP- down vs VAP+ |
| 208107_s_at | LOC81691 | exonuclease NEF-sp | 0.0414918 | 0.431824 | 1.14716 | VAP- up vs VAP+ |
| 204965_at | GC | group-specific component (vitamin D binding protein) | 0.041549 | 0.431824 | -1.13328 | VAP- down vs VAP+ |
| 200888_s_at | RPL23 | ribosomal protein L23 | 0.041578 | 0.431824 | 1.32751 | VAP- up vs VAP+ |
| 205219_s_at | GALK2 | galactokinase 2 | 0.041942 | 0.431824 | 1.08068 | VAP- up vs VAP+ |
| 220627_at | CST8 | cystatin 8 (cystatin-related epididymal specific) | 0.0420546 | 0.431824 | -1.13692 | VAP- down vs VAP+ |
| 211875_x_at | PCDHGA10 | protocadherin gamma subfamily A, 10 | 0.0420651 | 0.431824 | 1.1057 | VAP- up vs VAP+ |
| 200926_at | RPS23 | ribosomal protein S23 | 0.0420679 | 0.431824 | 1.30376 | VAP- up vs VAP+ |
| 221298_s_at | SLC22A8 | solute carrier family 22 (organic anion transporter), member 8 | 0.0421141 | 0.431824 | 1.10605 | VAP- up vs VAP+ |
| 200065_s_at | ARF1 | ADP-ribosylation factor 1 | 0.0421366 | 0.431824 | 1.15139 | VAP- up vs VAP+ |
| 208624_s_at | EIF4G1 | eukaryotic translation initiation factor 4 gamma, 1 | 0.0421594 | 0.431824 | -1.20325 | VAP- down vs VAP+ |
| 209211_at | KLF5 | Kruppel-like factor 5 (intestinal) | 0.0421714 | 0.431824 | -1.09509 | VAP- down vs VAP+ |
| 37965_at | PARVB | parvin, beta | 0.0422762 | 0.431824 | 1.26353 | VAP- up vs VAP+ |
| 219914_at | ECEL1 | endothelin converting enzyme-like 1 | 0.0423056 | 0.431824 | 1.11987 | VAP- up vs VAP+ |
| 217296_at | KIR2DL1 | Killer cell immunoglobulin-like receptor, two domains, long cytoplasmic tail, 1 | 0.0423062 | 0.431824 | 1.11471 | VAP- up vs VAP+ |
| 204424_s_at | LMO3 | LIM domain only 3 (rhombotin-like 2) | 0.0423312 | 0.431824 | -1.25866 | VAP- down vs VAP+ |
| 220160_s_at | KPTN | kaptin (actin binding protein) | 0.0424644 | 0.431824 | -1.18922 | VAP- down vs VAP+ |
| 211010_s_at | NCR3 | natural cytotoxicity triggering receptor 3 | 0.0425778 | 0.431824 | 1.13661 | VAP- up vs VAP+ |
| 221426_s_at | OR3A3 | olfactory receptor, family 3, subfamily A, member 3 | 0.0426116 | 0.431824 | 1.133 | VAP- up vs VAP+ |
| 219004_s_at | C21orf45 | chromosome 21 open reading frame 45 | 0.0426494 | 0.431824 | -1.11124 | VAP- down vs VAP+ |
| 219155_at | PITPNC1 | phosphatidylinositol transfer protein, cytoplasmic 1 | 0.0426704 | 0.431824 | 1.20499 | VAP- up vs VAP+ |
| 208726_s_at | EIF2S2 | eukaryotic translation initiation factor 2, subunit 2 beta, 38kDa | 0.0427335 | 0.431824 | 1.23288 | VAP- up vs VAP+ |
| 200809_x_at | RPL12 | ribosomal protein L12 | 0.0428004 | 0.431824 | 1.33176 | VAP- up vs VAP+ |
| 207953_at | --- | --- | 0.0428332 | 0.431824 | 1.26154 | VAP- up vs VAP+ |
| 208519_x_at | GNRH2 | gonadotropin-releasing hormone 2 | 0.0428455 | 0.431824 | 1.1628 | VAP- up vs VAP+ |
| 201145_at | HAX1 | HCLS1 associated protein X-1 | 0.0428845 | 0.431824 | 1.17868 | VAP- up vs VAP+ |
| 203327_at | IDE | insulin-degrading enzyme | 0.0428921 | 0.431824 | 1.1087 | VAP- up vs VAP+ |
| 204906_at | RPS6KA2 | ribosomal protein S6 kinase, 90kDa, polypeptide 2 | 0.0428964 | 0.431824 | 1.2077 | VAP- up vs VAP+ |
| 221033_s_at | RNF17 | ring finger protein 17 | 0.0429554 | 0.431824 | 1.13651 | VAP- up vs VAP+ |
| 206140_at | LHX2 | LIM homeobox 2 | 0.0430406 | 0.431824 | 1.09455 | VAP- up vs VAP+ |
| 204017_at | KDELR3 | KDEL (Lys-Asp-Glu-Leu) endoplasmic reticulum protein retention receptor 3 | 0.0431203 | 0.431824 | -1.1355 | VAP- down vs VAP+ |
| 208544_at | ADRA2B | adrenergic, alpha-2B-, receptor | 0.0431997 | 0.431824 | 1.13891 | VAP- up vs VAP+ |
| 209615_s_at | PAK1 | p21 protein (Cdc42/Rac)-activated kinase 1 | 0.0432087 | 0.431824 | 1.34397 | VAP- up vs VAP+ |
| 207073_at | CDKL2 | cyclin-dependent kinase-like 2 (CDC2-related kinase) | 0.0432212 | 0.431824 | -1.26503 | VAP- down vs VAP+ |
| 206691_s_at | PDIA2 | protein disulfide isomerase family A, member 2 | 0.0432445 | 0.431824 | 1.10275 | VAP- up vs VAP+ |
| 203074_at | ANXA8 /// ANXA8L1 /// ANXA8L2 | annexin A8 /// annexin A8-like 1 /// annexin A8-like 2 | 0.0432915 | 0.431824 | -1.13809 | VAP- down vs VAP+ |
| 207048_at | SLC6A11 | solute carrier family 6 (neurotransmitter transporter, GABA), member 11 | 0.0433509 | 0.431824 | 1.12231 | VAP- up vs VAP+ |
| 201327_s_at | CCT6A | chaperonin containing TCP1, subunit 6A (zeta 1) | 0.0433634 | 0.431824 | 1.25387 | VAP- up vs VAP+ |
| 204998_s_at | ATF5 | activating transcription factor 5 | 0.0433986 | 0.431824 | 1.33522 | VAP- up vs VAP+ |
| 221110_x_at | PDE11A | phosphodiesterase 11A | 0.0434377 | 0.431824 | -1.18654 | VAP- down vs VAP+ |
| 207519_at | SLC6A4 | solute carrier family 6 (neurotransmitter transporter, serotonin), member 4 | 0.0434435 | 0.431824 | -1.09121 | VAP- down vs VAP+ |
| 201250_s_at | SLC2A1 | solute carrier family 2 (facilitated glucose transporter), member 1 | 0.0434449 | 0.431824 | 1.09212 | VAP- up vs VAP+ |
| 220423_at | PLA2G2D | phospholipase A2, group IID | 0.0435943 | 0.431824 | -1.14549 | VAP- down vs VAP+ |
| 217932_at | MRPS7 | mitochondrial ribosomal protein S7 | 0.0436735 | 0.431824 | 1.15327 | VAP- up vs VAP+ |
| 214198_s_at | DGCR2 | DiGeorge syndrome critical region gene 2 | 0.0437033 | 0.431824 | -1.37061 | VAP- down vs VAP+ |
| 206910_x_at | CFHR2 | complement factor H-related 2 | 0.0437785 | 0.431824 | 1.0712 | VAP- up vs VAP+ |
| 220888_s_at | CASS4 | Cas scaffolding protein family member 4 | 0.0438152 | 0.431824 | -1.10334 | VAP- down vs VAP+ |
| 220183_s_at | NUDT6 | nudix (nucleoside diphosphate linked moiety X)-type motif 6 | 0.043939 | 0.431824 | 1.07566 | VAP- up vs VAP+ |
| 200000_s_at | PRPF8 | PRP8 pre-mRNA processing factor 8 homolog (S. cerevisiae) | 0.043941 | 0.431824 | 1.21179 | VAP- up vs VAP+ |
| 220948_s_at | ATP1A1 | ATPase, Na+/K+ transporting, alpha 1 polypeptide | 0.0439808 | 0.431824 | 1.24218 | VAP- up vs VAP+ |
| 205261_at | PGC | progastricsin (pepsinogen C) | 0.0440664 | 0.431824 | 1.08068 | VAP- up vs VAP+ |
| 201578_at | PODXL | podocalyxin-like | 0.0440712 | 0.431824 | 1.09113 | VAP- up vs VAP+ |
| 203727_at | SKIV2L | superkiller viralicidic activity 2-like (S. cerevisiae) | 0.0441879 | 0.431824 | 1.16099 | VAP- up vs VAP+ |
| 218475_at | TRMT2A | TRM2 tRNA methyltransferase 2 homolog A (S. cerevisiae) | 0.0442149 | 0.431824 | 1.10613 | VAP- up vs VAP+ |
| 202910_s_at | CD97 | CD97 molecule | 0.044258 | 0.431824 | 1.30538 | VAP- up vs VAP+ |
| 200705_s_at | EEF1B2 | eukaryotic translation elongation factor 1 beta 2 | 0.0442714 | 0.431824 | 1.36751 | VAP- up vs VAP+ |
| 200094_s_at | EEF2 | eukaryotic translation elongation factor 2 | 0.0443805 | 0.431824 | 1.34085 | VAP- up vs VAP+ |
| 208775_at | XPO1 | exportin 1 (CRM1 homolog, yeast) | 0.0444115 | 0.431824 | 1.25105 | VAP- up vs VAP+ |
| 205199_at | CA9 | carbonic anhydrase IX | 0.0444963 | 0.431824 | -1.22823 | VAP- down vs VAP+ |
| 200674_s_at | RPL32 | ribosomal protein L32 | 0.0445183 | 0.431824 | 1.3475 | VAP- up vs VAP+ |
| 205966_at | TAF13 | TAF13 RNA polymerase II, TATA box binding protein (TBP)-associated factor, 18kDa | 0.0445391 | 0.431824 | -1.04257 | VAP- down vs VAP+ |
| 200060_s_at | RNPS1 | RNA binding protein S1, serine-rich domain | 0.0445668 | 0.431824 | 1.21025 | VAP- up vs VAP+ |
| 220214_at | ZNF215 | zinc finger protein 215 | 0.0446335 | 0.431824 | -1.11123 | VAP- down vs VAP+ |
| 212867_at | --- | --- | 0.0446967 | 0.431824 | -1.33074 | VAP- down vs VAP+ |
| 204247_s_at | CDK5 | cyclin-dependent kinase 5 | 0.0447445 | 0.431824 | 1.11708 | VAP- up vs VAP+ |
| 200912_s_at | EIF4A2 | eukaryotic translation initiation factor 4A2 | 0.0447568 | 0.431824 | 1.27457 | VAP- up vs VAP+ |
| 210908_s_at | PFDN5 | prefoldin subunit 5 | 0.0447772 | 0.431824 | 1.32354 | VAP- up vs VAP+ |
| 208887_at | EIF3G | eukaryotic translation initiation factor 3, subunit G | 0.0447939 | 0.431824 | 1.26386 | VAP- up vs VAP+ |
| 204673_at | MUC2 | mucin 2, oligomeric mucus/gel-forming | 0.0448012 | 0.431824 | -1.09853 | VAP- down vs VAP+ |
| 220768_s_at | CSNK1G3 | casein kinase 1, gamma 3 | 0.0449015 | 0.432205 | -1.23757 | VAP- down vs VAP+ |
| 204067_at | SUOX | sulfite oxidase | 0.0450187 | 0.432369 | -1.17665 | VAP- down vs VAP+ |
| 208510_s_at | PPARG | peroxisome proliferator-activated receptor gamma | 0.045105 | 0.432369 | -1.10703 | VAP- down vs VAP+ |
| 206090_s_at | DISC1 /// TSNAX-DISC1 | disrupted in schizophrenia 1 /// TSNAX-DISC1 gene | 0.0452582 | 0.432369 | -1.16984 | VAP- down vs VAP+ |
| 210852_s_at | AASS | aminoadipate-semialdehyde synthase | 0.0453216 | 0.432369 | -1.14139 | VAP- down vs VAP+ |
| 215148_s_at | APBA3 | amyloid beta (A4) precursor protein-binding, family A, member 3 | 0.0453783 | 0.432369 | 1.21875 | VAP- up vs VAP+ |
| 206047_at | GNB3 | guanine nucleotide binding protein (G protein), beta polypeptide 3 | 0.0454098 | 0.432369 | 1.12545 | VAP- up vs VAP+ |
| 212455_at | YTHDC1 | YTH domain containing 1 | 0.0454325 | 0.432369 | 1.30351 | VAP- up vs VAP+ |
| 208155_x_at | GAGE1 /// GAGE12F /// GAGE12G /// GAGE12I /// GAGE12J /// GAGE4 /// GAGE5 /// GAGE6 /// GAGE7 | G antigen 1 /// G antigen 12F /// G antigen 12G /// G antigen 12I /// G antigen | 0.0454811 | 0.432369 | -1.11656 | VAP- down vs VAP+ |
| 218619_s_at | SUV39H1 | suppressor of variegation 3-9 homolog 1 (Drosophila) | 0.0455257 | 0.432369 | 1.10107 | VAP- up vs VAP+ |
| 34187_at | RBMS2 | RNA binding motif, single stranded interacting protein 2 | 0.0456165 | 0.432369 | 1.05904 | VAP- up vs VAP+ |
| 210133_at | CCL11 | chemokine (C-C motif) ligand 11 | 0.0456296 | 0.432369 | -1.23428 | VAP- down vs VAP+ |
| 214623_at | FBXW4P1 | F-box and WD repeat domain containing 4 pseudogene 1 | 0.045648 | 0.432369 | -1.11183 | VAP- down vs VAP+ |
| 209477_at | EMD | emerin | 0.0458654 | 0.433035 | 1.32475 | VAP- up vs VAP+ |
| 200096_s_at | ATP6V0E1 | ATPase, H+ transporting, lysosomal 9kDa, V0 subunit e1 | 0.046243 | 0.433035 | -1.26228 | VAP- down vs VAP+ |
| 220277_at | CXXC4 | CXXC finger 4 | 0.0464332 | 0.433035 | -1.08245 | VAP- down vs VAP+ |
| 214452_at | BCAT1 | branched chain amino-acid transaminase 1, cytosolic | 0.0464737 | 0.433035 | 1.29367 | VAP- up vs VAP+ |
| 205531_s_at | GLS2 | glutaminase 2 (liver, mitochondrial) | 0.0465583 | 0.433035 | 1.09319 | VAP- up vs VAP+ |
| 204819_at | FGD1 | FYVE, RhoGEF and PH domain containing 1 | 0.0465732 | 0.433035 | -1.12351 | VAP- down vs VAP+ |
| 217766_s_at | TMEM50A | transmembrane protein 50A | 0.0465861 | 0.433035 | -1.32699 | VAP- down vs VAP+ |
| 206440_at | LIN7A | lin-7 homolog A (C. elegans) | 0.0466072 | 0.433035 | -1.27558 | VAP- down vs VAP+ |
| 206111_at | RNASE2 | ribonuclease, RNase A family, 2 (liver, eosinophil-derived neurotoxin) | 0.0466172 | 0.433035 | -1.50365 | VAP- down vs VAP+ |
| 218266_s_at | NCS1 | neuronal calcium sensor 1 | 0.0466932 | 0.433035 | 1.10592 | VAP- up vs VAP+ |
| 205059_s_at | IDUA | iduronidase, alpha-L- | 0.0466955 | 0.433035 | 1.12339 | VAP- up vs VAP+ |
| 203677_s_at | TARBP2 | TAR (HIV-1) RNA binding protein 2 | 0.0466996 | 0.433035 | 1.14609 | VAP- up vs VAP+ |
| 219922_s_at | LTBP3 | latent transforming growth factor beta binding protein 3 | 0.0467492 | 0.433035 | 1.15808 | VAP- up vs VAP+ |
| 205479_s_at | PLAU | plasminogen activator, urokinase | 0.0467715 | 0.433035 | 2.08935 | VAP- up vs VAP+ |
| 221065_s_at | CHST8 | carbohydrate (N-acetylgalactosamine 4-0) sulfotransferase 8 | 0.0467723 | 0.433035 | 1.13052 | VAP- up vs VAP+ |
| 215918_s_at | SPTBN1 | spectrin, beta, non-erythrocytic 1 | 0.0467796 | 0.433035 | -1.18719 | VAP- down vs VAP+ |
| 32088_at | BLZF1 | basic leucine zipper nuclear factor 1 | 0.0467902 | 0.433035 | 1.03743 | VAP- up vs VAP+ |
| 204937_s_at | ZNF274 | zinc finger protein 274 | 0.0468141 | 0.433035 | 1.34697 | VAP- up vs VAP+ |
| 49485_at | PRDM4 | PR domain containing 4 | 0.0470201 | 0.434376 | -1.07735 | VAP- down vs VAP+ |
| 203871_at | SENP3 | SUMO1/sentrin/SMT3 specific peptidase 3 | 0.0472306 | 0.435226 | 1.15471 | VAP- up vs VAP+ |
| 204133_at | RRP9 | ribosomal RNA processing 9, small subunit (SSU) processome component, homolog (y | 0.0473051 | 0.435226 | 1.11409 | VAP- up vs VAP+ |
| 221379_at | --- | --- | 0.0473437 | 0.435226 | 1.12224 | VAP- up vs VAP+ |
| 204587_at | SLC25A14 | solute carrier family 25 (mitochondrial carrier, brain), member 14 | 0.0474306 | 0.435226 | -1.22327 | VAP- down vs VAP+ |
| 218729_at | LXN | latexin | 0.0474562 | 0.435226 | -1.09689 | VAP- down vs VAP+ |
| 218350_s_at | GMNN | geminin, DNA replication inhibitor | 0.0475805 | 0.435226 | -1.07708 | VAP- down vs VAP+ |
| 201500_s_at | PPP1R11 | protein phosphatase 1, regulatory (inhibitor) subunit 11 | 0.0476189 | 0.435226 | 1.17599 | VAP- up vs VAP+ |
| 37996_s_at | DMPK | dystrophia myotonica-protein kinase | 0.0477193 | 0.435226 | 1.12521 | VAP- up vs VAP+ |
| 213606_s_at | ARHGDIA | Rho GDP dissociation inhibitor (GDI) alpha | 0.0477729 | 0.435226 | -1.64737 | VAP- down vs VAP+ |
| 203816_at | DGUOK | deoxyguanosine kinase | 0.0477961 | 0.435226 | 1.16347 | VAP- up vs VAP+ |
| 204908_s_at | BCL3 | B-cell CLL/lymphoma 3 | 0.0478529 | 0.435226 | 1.2541 | VAP- up vs VAP+ |
| 211956_s_at | EIF1 | eukaryotic translation initiation factor 1 | 0.0478625 | 0.435226 | 1.12217 | VAP- up vs VAP+ |
| 202045_s_at | GRLF1 | glucocorticoid receptor DNA binding factor 1 | 0.0479075 | 0.435226 | 1.12658 | VAP- up vs VAP+ |
| 221131_at | A4GNT | alpha-1,4-N-acetylglucosaminyltransferase | 0.0481651 | 0.435711 | 1.08777 | VAP- up vs VAP+ |
| 201094_at | RPS29 | ribosomal protein S29 | 0.0481748 | 0.435711 | 1.27548 | VAP- up vs VAP+ |
| 209500_x_at | TNFSF12-TNFSF13 /// TNFSF13 | TNFSF12-TNFSF13 readthrough /// tumor necrosis factor (ligand) superfamily, memb | 0.0483026 | 0.435711 | -1.27615 | VAP- down vs VAP+ |
| 205476_at | CCL20 | chemokine (C-C motif) ligand 20 | 0.0483617 | 0.435711 | 1.26402 | VAP- up vs VAP+ |
| 208161_s_at | ABCC3 | ATP-binding cassette, sub-family C (CFTR/MRP), member 3 | 0.0483962 | 0.435711 | -1.2002 | VAP- down vs VAP+ |
| 202230_s_at | CHERP | calcium homeostasis endoplasmic reticulum protein | 0.048421 | 0.435711 | 1.34621 | VAP- up vs VAP+ |
| 204909_at | DDX6 | DEAD (Asp-Glu-Ala-Asp) box polypeptide 6 | 0.0484687 | 0.435711 | -1.32265 | VAP- down vs VAP+ |
| 213650_at | GOLGA8A /// GOLGA8B | golgin A8 family, member A /// golgin A8 family, member B | 0.0485959 | 0.435711 | 1.10999 | VAP- up vs VAP+ |
| 208764_s_at | ATP5G2 | ATP synthase, H+ transporting, mitochondrial F0 complex, subunit C2 (subunit 9) | 0.0486621 | 0.435711 | 1.29606 | VAP- up vs VAP+ |
| 206592_s_at | AP3D1 | adaptor-related protein complex 3, delta 1 subunit | 0.0486661 | 0.435711 | 1.18166 | VAP- up vs VAP+ |
| 205041_s_at | ORM1 /// ORM2 | orosomucoid 1 /// orosomucoid 2 | 0.048764 | 0.435711 | 2.21107 | VAP- up vs VAP+ |
| 201387_s_at | UCHL1 | ubiquitin carboxyl-terminal esterase L1 (ubiquitin thiolesterase) | 0.0487725 | 0.435711 | -1.12856 | VAP- down vs VAP+ |
| 203618_at | FAIM2 | Fas apoptotic inhibitory molecule 2 | 0.0487881 | 0.435711 | 1.1267 | VAP- up vs VAP+ |
| 216092_s_at | SLC7A8 | solute carrier family 7 (amino acid transporter, L-type), member 8 | 0.0488184 | 0.435711 | 1.12717 | VAP- up vs VAP+ |
| 203984_s_at | CASP9 | caspase 9, apoptosis-related cysteine peptidase | 0.0490181 | 0.436945 | 1.15457 | VAP- up vs VAP+ |
| 207269_at | DEFA4 | defensin, alpha 4, corticostatin | 0.0492372 | 0.437606 | -1.97243 | VAP- down vs VAP+ |
| 219210_s_at | RAB8B | RAB8B, member RAS oncogene family | 0.0493102 | 0.437606 | 1.1929 | VAP- up vs VAP+ |
| 209295_at | TNFRSF10B | tumor necrosis factor receptor superfamily, member 10b | 0.0493758 | 0.437606 | 1.37832 | VAP- up vs VAP+ |
| 206735_at | CHRNA4 | cholinergic receptor, nicotinic, alpha 4 | 0.0494911 | 0.437606 | 1.09801 | VAP- up vs VAP+ |
| 210565_at | GCGR | glucagon receptor | 0.0494918 | 0.437606 | 1.12779 | VAP- up vs VAP+ |
| 200854_at | NCOR1 | nuclear receptor co-repressor 1 | 0.0494989 | 0.437606 | -1.20384 | VAP- down vs VAP+ |
| 210817_s_at | CALCOCO2 | calcium binding and coiled-coil domain 2 | 0.0495794 | 0.437606 | 1.18064 | VAP- up vs VAP+ |
| 220620_at | CRCT1 | cysteine-rich C-terminal 1 | 0.0495844 | 0.437606 | -1.18404 | VAP- down vs VAP+ |
| 36019_at | STK19 | serine/threonine kinase 19 | 0.0498739 | 0.438895 | 1.10263 | VAP- up vs VAP+ |
| 221343_at | OR11A1 | olfactory receptor, family 11, subfamily A, member 1 | 0.0499572 | 0.438895 | -1.08684 | VAP- down vs VAP+ |
| 200038_s_at | RPL17 | ribosomal protein L17 | 0.0499648 | 0.438895 | 1.29154 | VAP- up vs VAP+ |
| 200971_s_at | SERP1 | stress-associated endoplasmic reticulum protein 1 | 0.0499773 | 0.438895 | 1.15258 | VAP- up vs VAP+ |
