# Supplementary material for: Developing a Gene Expression Model for Predicting Ventilator-Associated Pneumonia in Trauma Patients: A Pilot Study
Source: PLoS One. 2012 Aug 15;7(8):e42065. doi: 10.1371/journal.pone.0042065 (PMC3419717; doi:10.1371/journal.pone.0042065)
Supplement: Table S2 — Significantly enriched Gene Ontology categories. This table contains only the most distal (specific) categories in a particular ontology. The number of genes within each category that are either up- or down-regulated in VAP+ patients is indicated. For a complete listing of all enriched GO categories, please see Table S1. (DOC) [file pone.0042065.s002.doc]

Table S2. Gene Ontology categories that were significantly enriched in the subset of differentially expressed genes with respect to VAP. This table contains only the most distal (specific) categories in a particular ontology. The number of genes within each category that are either up- or down-regulated in VAP+ patients is indicated. For a complete listing of all enriched GO categories, please see Table S1.

|  | **Functional Category** | **VAP+ Down** | **VAP+ Up** | **p-value** |
| --- | --- | --- | --- | --- |
| GO Biological process | |  |  |  |
|  | translational elongation | 25 | 0 | 5.71E-08 |
|  | DNA unwinding during replication | 3 | 2 | 3.00E-04 |
|  | protein folding | 17 | 4 | 5.00E-04 |
|  | pyruvate biosynthetic process | 1 | 2 | 8.00E-04 |
|  | glyoxylate cycle | 1 | 1 | 8.80E-03 |
|  | cellular amino acid metabolic process | 18 | 7 | 6.70E-03 |
|  | antimicrobial humoral response | 2 | 0 | 8.80E-03 |
| GO Molecular function | |  |  |  |
|  | structural constituent of ribosome | 25 | 0 | 6.40E-08 |
|  | 1-alkyl-2-acetylglycerophosphocholine esterase activity | 2 | 1 | 3.10E-03 |
|  | bacterial binding | 0 | 5 | 1.00E-03 |
|  | endopeptidase inhibitor activity | 12 | 6 | 2.30E-03 |
|  | metalloendopeptidase inhibitor activity | 2 | 2 | 4.00E-03 |
|  | serine-type endopeptidase activity | 14 | 5 | 2.00E-03 |
|  | metalloenzyme regulator activity | 2 | 2 | 4.00E-03 |
| GO Cellular component | |  |  |  |
|  | ribonucleoprotein complex | 43 | 2 | 8.64E-05 |
|  | cytosolic large ribosomal subunit | 10 | 0 | 2.00E-04 |
|  | chaperonin-containing T-complex | 4 | 0 | 3.00E-04 |
|  | cytosolic small ribosomal subunit | 7 | 2 | 1.20E-03 |
| KEGG category | |  |  |  |
|  | Ribosome | 24 | 0 | 1.24E-08 |
|  | Vibrio cholerae infection | 7 | 3 | 3.50E-03 |
|  | Glycine | 4 | 3 | 4.90E-03 |
